# Lomitapide enhances cytotoxic effects of temozolomide in chemotherapy-resistant glioblastoma

**DOI:** 10.1172/jci.insight.186703

**Published:** 2025-07-22

**Authors:** Alyona Ivanova, Taylor M. Wilson, Kimia Ghannad-Zadeh, Esmond Tse, Robert Flick, Megan Wu, Sunit Das

**Affiliations:** 1 The Arthur and Sonia Labatt Brain Tumor Research Center, The Hospital for Sick Children; Toronto, Canada.; 2 Institute of Medical Sciences,; 3 Department of Chemical Engineering and Applied Chemistry,; 4 Division of Neurosurgery, and; 5 Keenan Chair in Surgery, St. Michael’s Hospital, University of Toronto, Toronto, Ontario, Canada.

**Keywords:** Cell biology, Oncology, Brain cancer, Clinical practice, Drug therapy

## Abstract

More than a third of patients with glioblastoma experience tumor progression during adjuvant therapy. In this study, we performed a high-throughput drug repurposing screen of FDA-approved agents capable of crossing the blood-brain barrier in order to find agents to counteract acquired or inherent glioma cell resistance to temozolomide-associated cytotoxicity. We identified the cholesterol processing inhibitor, lomitapide, as a potential chemosensitizer in glioblastoma. In vitro treatment of temozolomide-resistant glioblastoma cells with lomitapide resulted in decreased intracellular ubiquinone levels and sensitized cells to temozolomide-induced ferroptosis. Concomitant treatment with lomitapide and temozolomide (TMZ) prolonged survival and delayed tumor recurrence in a mouse glioblastoma model, compared with treatment xwith TMZ alone. Our data identified lomitapide as a potential adjunct for treatment of temozolomide-resistant glioblastoma.

## Abstract 2

Cholesterol-lowering drug lomitapide can enhance the effectiveness of temozolomide chemotherapy in treating chemo-resistant glioblastoma by increasing cancer cell sensitivity to treatment.

## Graphical abstract

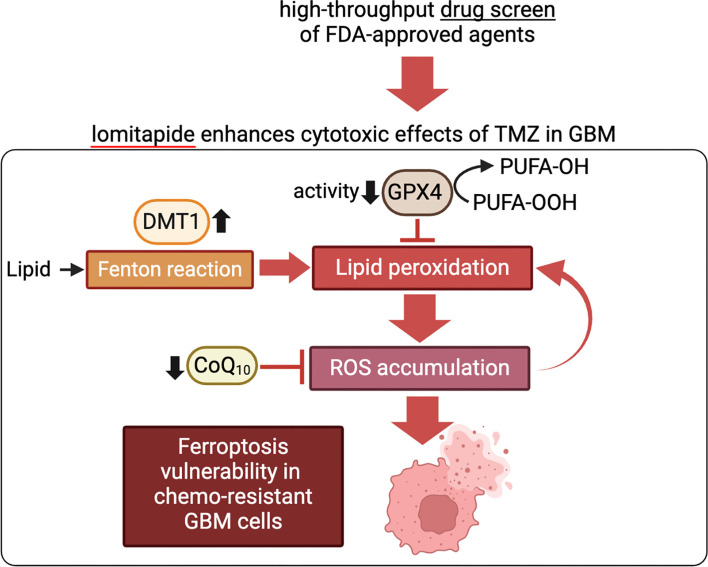

## Introduction

Glioblastoma is the most prevalent and aggressive primary brain tumor in adults (1). The current standard of care for patients with glioblastoma includes maximal safe surgical resection followed by radiation and chemotherapy with the alkylating agent, temozolomide (TMZ). While the addition of TMZ to existing conventional treatment has resulted in a significant increase in the number of long-term survivors (2), more than one-third of patients experience tumor progression during adjuvant therapy (3), suggesting that many of these patients harbor tumor cells that are intrinsically resistant to TMZ-associated cytotoxicity. There is substantial need for novel therapies to complement or improve our current treatments.

Drug repurposing has gained attention in cancer research for its time- and monetary efficiency in advancing chemical leads for clinical studies (4). Consideration of FDA-approved agents for a novel indication substantially decreases the time required for agents to go from bench to bedside compared with that required for existing agents, as toxicological data for these drugs is publicly available (5).

Using a high-throughput drug screen of FDA-approved agents, we identified 8 agents that were predicted to have good brain penetration and that exhibited cytotoxicity against glioma cells when combined with TMZ. As multiple recent reports have identified cholesterol biosynthesis as a vulnerability in glioma cells (6–11), we chose to investigate the lipid-lowering drug, lomitapide (Juxtapid) (12–13), as a potential adjunct treatment for glioblastoma. We find that lomitapide inhibits the mevalonate pathway of de novo cholesterol biosynthesis in TMZ-resistant glioma cells, resulting in cholesterol and ubiquinone (CoQ10) deficiency. Depletion of antioxidant CoQ10, in turn, causes excessive accumulation of cellular reactive oxygen species (ROS) that prime TMZ-resistant glioma cells for ferroptosis. In a mouse xenograft model, concurrent treatment with lomitapide and TMZ significantly delays tumor recurrence and prolongs survival, compared with treatment with TMZ alone. Our findings identify lomitapide as a potential therapeutic agent capable of targeting treatment resistance and delaying tumor progression in glioblastoma.

## Results

### Identification of lomitapide through a high-throughput drug screen.

Cellular resistance to the cytotoxic effects of TMZ is a fundamental cause of treatment failure and disease recurrence in patients with glioblastoma (1–3, 14). We have previously reported on TMZ-resistant glioblastoma cells (TR-U251), a TMZ-resistant U251 glioblastoma cell line that we developed through serial exposure to increasing concentrations of TMZ (15). Using this model, we sought to identify agents capable of targeting TMZ-resistant glioblastoma cells.

**Figure 1:**
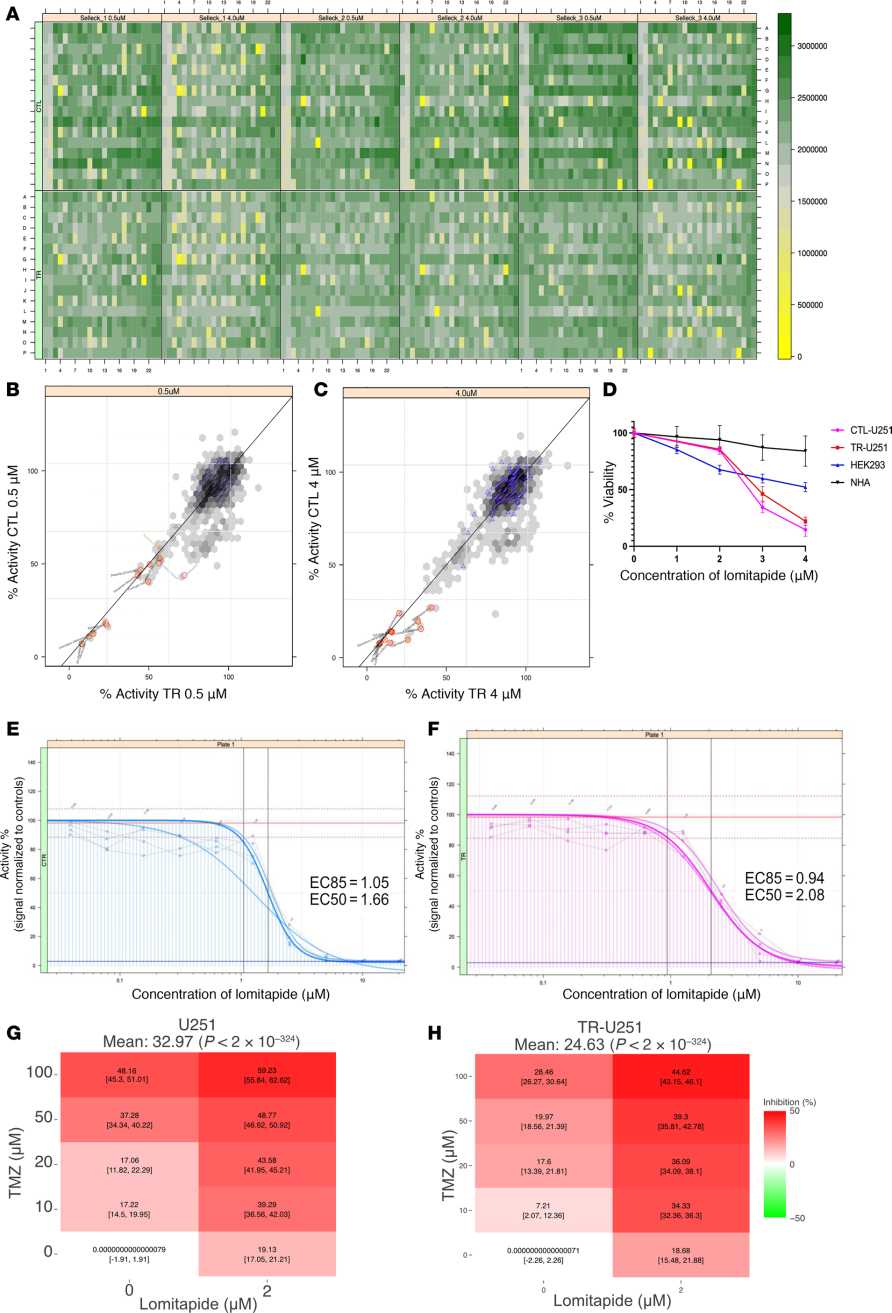
High-throughput screening reveals FDA-approved agents capable of inducing toxicity in glioblastoma cell lines. (**A**) Heat map of CTL- and TR-U251 cell viability following screening with SelleckChem’s FDA-approved chemical library at 0.5 and 4.0 μM concentrations. Results in the low-density (yellow) end were evaluated as potential drug hits and subjected to further analysis. Correlation plot of CTL- and TR-U251 results at 0.5 μM (**B**) and 4.0 μM (**C**) drug concentrations. Red circles represent 1% hits on the CTL-U251, orange crosses are indicative of 1% hits on TR-U251 cells, and blue triangles denote agents capable of crossing the BBB. (**D**) Viability assay shows CTL-U251, TR-U251, HEK-293, NHA cell response after treatment with serially increasing concentrations of lomitapide. The data is normalized to untreated control. Dose-response curve for CTL-U251 (**E**) and TR-U251 (**F**) cells treated with lomitapide. Dose-response curves were used to determine IC_50_ values for in vitro analysis. Dose-response matrix map for synergistic drug combination (lomitapide and TMZ) generated using SynergyFinder for (**G**) CTL-U251 and (**H**) TR-U251.

First, we performed a high-throughput drug screen using a library of approximately 900 FDA-approved candidates in the presence of TMZ. CTL- and TR-U251 cells were subjected to 72-hour screening with the library at 2 drug concentrations (0.5 and 4.0 μM), with DMSO used as a negative control (Figure 1A). Cell viability was used to identify potential hits. Thirty-one agents were found to result in cell death in both parental and TMZ-resistant U251 cells (Figure 1, B and C). Of these agents, analysis identified 8 as capable of crossing the blood-brain barrier (BBB): pralatrexate, dronedarone, lomitapide, ethacridine lactate monohydrate, delanzomib, omipalisib, tivantinib, and raltitrexed (Supplemental Table 1; supplemental material available online with this article; https://doi.org/10.1172/jci.insight.186703DS1). Only the lipid-lowering agent lomitapide (Supplemental Figure 1) produced a dose-response relationship in an exploitable dosage range and was selected for more stringent testing and optimal dose determination.

### Lomitapide exerts cytotoxic effects on glioma cells and sensitizes them to the effects of temozolomide.

To assess the cytotoxic drug response of lomitapide in glioma cells, a dose-response viability curve was constructed for CTL- and TR-U251 and cell lines treated with lomitapide for 72 hours. Lomitapide treatment resulted in cell death in both CTL-U251 and TR-U251 cell lines in a dose-dependent manner, with minimal effect on viability of normal HEK293 cells and NHA cells (Figure 1D). IC_50_ values were higher for the TR-U251 cell line (IC_50_ = 2.08 μM) than for the CTL-U251 line (IC_50_ = 1.66 μM), suggesting that resistance to cell death in TMZ-resistant cells is multifactorial in nature (Figure 1, E and F). We used SynergyFinder (16) to evaluate drug combination synergy. The combination of lomitapide and TMZ demonstrated significant synergy across multiple synergy models (Figure 1, G and H). The Loewe, Bliss, HSA, and ZIP scores were 16.38, 4.15, 16.63, and 4.2, respectively, for CTL-U251, and 16.64, 4.61, 17.01, and 4.58, respectively, for TR-U251 cells (Figure 1, G and H, and Supplemental Figure 2).

**Figure 2:**
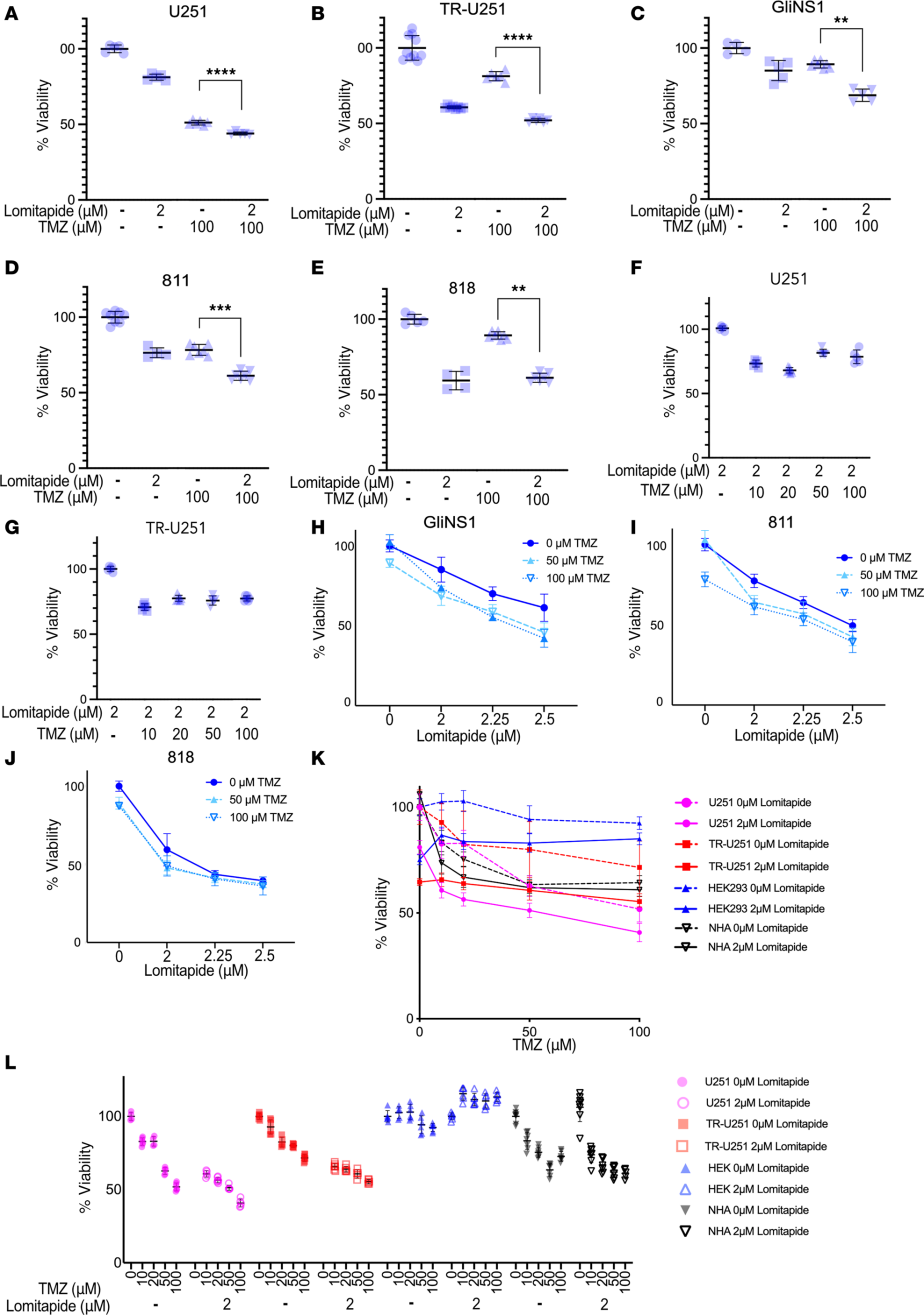
Lomitapide targets GBM cells and sensitizes them to the effects of TMZ, while minimizing damage to normal cells. Cytotoxicity estimated at 72 hours after treatment by Cell Viability Assay. Compared with TMZ alone, CTL-(**A**) and TR-U251 (**B**) cell lines treated concomitantly with lomitapide and TMZ had significantly decreased cell viability. Concentration of lomitapide near its IC_80_ value added in combination with TMZ significantly reduces cell viability, compared with TMZ treatment alone in GSC lines GliNS1 (**C**), 811 (**D**), 818 (**E**). CTL-U251 (**F**), TR-U251 (**G**), GliNS1 (**H**), 811 (**I**), 818 (**J**) cells treated with doses of TMZ 0–100 μM with or without lomitapide. Cell viability normalized to 2 μM lomitapide treatment without TMZ for CTL-U251 and TR-U251 cells. Data in **A**–**G** is presented as individual measurements with mean ± SD. Data is normalized to untreated control, mean ± SD (**H**–**J**). (**K** and **L**) Dose-response curves. Data is represented as mean ± SD (**K**), individual measurements (**L**) are normalized to untreated control with mean ± SD. 2-tailed Student’s *t* tests were used for statistical comparisons between 2 groups. **P < 0.01; ***P < 0.001; ****P < 0.0001.

We then tested lomitapide in vitro as an adjunct treatment with TMZ. CTL-U251 and TR-U251 cells were analyzed as 4 treatment groups: (a) no treatment; (b) 2 μM lomitapide (IC_80_); (c) 100 μM TMZ; or (d) concomitant lomitapide and TMZ. TR-U251 cells showed near complete resistance to single agent TMZ at all tested concentrations (0–100 μM) (Figure 2 K and L). In contrast, concomitant treatment with lomitapide and TMZ resulted in a statistically significant decrease in viability in both U251 (Figure 2A) and TR-U251 cells (Figure 2B) compared with treatment with TMZ alone. Notably, concomitant treatment with lomitapide greatly enhanced the cytotoxic effect of low-dose TMZ on CTL-U251 and TR-U251 cells, with equivalent effects seen at 10 μM of TMZ, compared with 100 μM (Figure 2, F, G, and L).

As glioma stem cells (GSCs) have been postulated to be the drivers of tumor progression and treatment resistance, we next examined the effect of lomitapide in multiple GSC cell lines. Briefly, three GSC lines (GliNS1, 811, and 818) were treated with lomitapide alone, TMZ alone, or concomitant lomitapide and TMZ, for 7 days. Concomitant treatment with lomitapide and TMZ resulted in a statistically significant decrease in cell viability in all 3 GSC lines, compared with treatment with single-agent TMZ (Figure 2, C–E and H–J).

We then investigated whether lomitapide exerts cytotoxic damage on normal cells. Concurrent treatment with lomitapide and TMZ resulted in reduced viability in both CTL-U251 and TR-251 cells but had no effect on viability in HEK293 or NHA cells (Figure 2, K and L).

**Figure 3:**
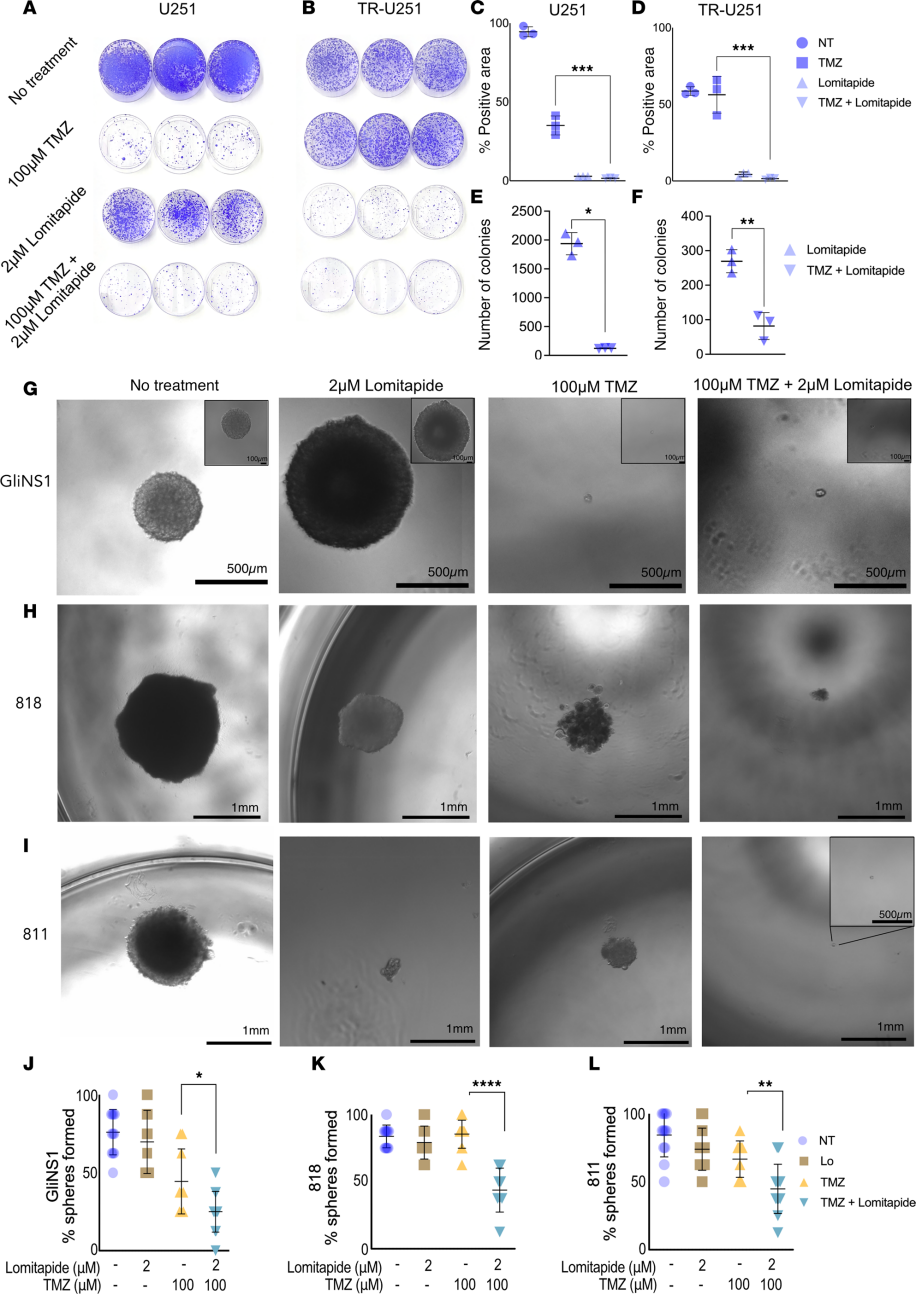
Lomitapide treatment combined with TMZ affects the ability of U251 and Tr-U251 cells to form colonies and reduces self-renewal capacity of GSCs. Crystal violet staining depicting colony formation following 100 μM TMZ, 2 μM lomitapide, or combination treatment in U251 (**A**) and TR-U251 cells (**B**). Cells were treated for 7 days, followed by 7 days of recovery in drug-free media. Three images representative for each condition are shown. Quantification of colony area (**C** and **D**) or counts (**E** and **F**) by ImageJ. Error bars indicate SD (*n* = 5). Sphere formation assays on GliNS1 (**G**), 818 (**H**), and 818 (**I**) cells treated with 2 μM lomitapide, 100 μM TMZ, or concomitantly with lomitapide and TMZ for 72 hours. After sorting, single cells were recovered in drug-free media for 4 weeks. Representative bright-field images are shown. Scale bar: 500 μm and 100 μm for GliNS1; 1mm for 818 cells, 1mm and 500 μm for 811. Percentage of spheres formed from GliNS1 (**J**), 811 (**K**), 818 (**L**) cells after 4 weeks. Data represents % of spheres formed / row of a 96-well plate with mean ± SD (*n* = 8). Two-tailed Student’s *t* tests were used for statistical comparisons between 2 groups. *P < 0.05; **P < 0.01; ***P < 0.001.

U251 and TR-U251 cells treated with TMZ, lomitapide, or combinatory treatment, following a brief recovery in media, were then assayed for colony formation. Crystal violet staining revealed a significant reduction in the ability of U251 glioma cells to form colonies following treatment with the combination of lomitapide and TMZ compared with U251 cells treated with TMZ alone (Figure 3, A–F). The negative effect of combinatory treatment was even more significant on TR-U251 cells (Figure 3, B, D, and F).

To examine whether lomitapide treatment combined with TMZ affected the self-renewal capacity of GSCs, we conducted a nonadherent sphere assay with GSCs following pretreatment with control media, lomitapide alone, TMZ alone, or concomitant lomitapide and TMZ for 72 hours, followed by recovery of single cells in drug-free media. Cells pretreated with combination therapy showed a significant reduction in sphere formation (Figure 3, G–L) and formed much smaller spheres compared with cells pretreated with TMZ or lomitapide alone (Figure 3, G–I).

### Lomitapide inhibits de novo cholesterol synthesis.

**Figure 4:**
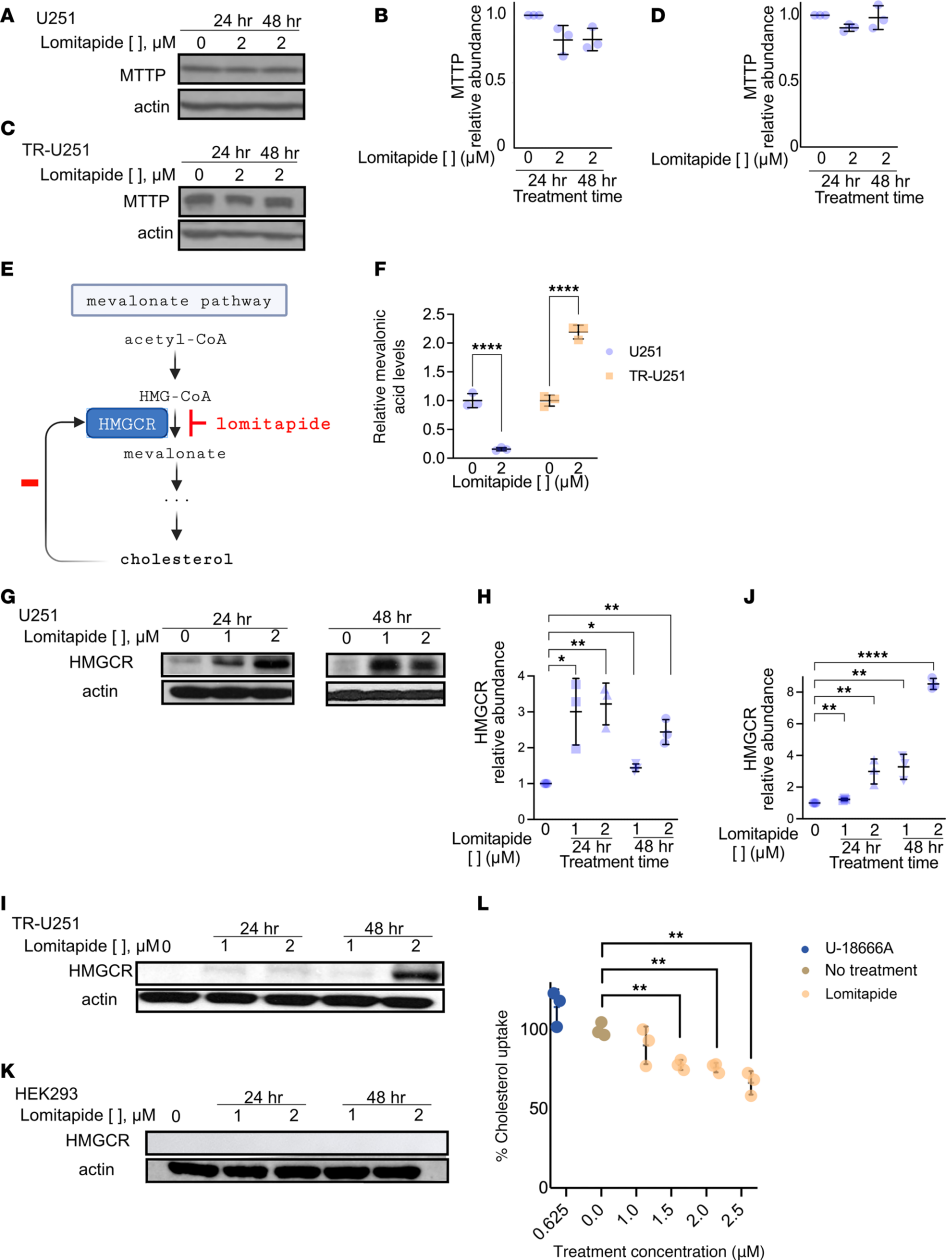
Lomitapide cytotoxicity in vitro is attributed to inhibition of de novo cholesterol synthesis. Lomitapide (2 μM) treatment has no effect on the expression of microsomal triglyceride transport protein (MTTP) in CTL-U251 (**A** and **B**) and TR-U251 (**C** and **D**) cells as confirmed by Western blot analysis after 24 and 48 hours of drug treatment. (**E**) Proposed mechanism of action of lomitapide. Lomitapide inhibits the critical rate-limiting step of mevalonate pathway by binding to HMGCR. While normally, cholesterol production acts through a negative feedback loop to control HMGCR synthesis, reduction in cholesterol production causes accumulation of HMGCR. (**F**) Targeted LC-MS results showing relative concentration of mevalonic acid in CTL-U251 cells treated with 2 μM lomitapide for 72 hours (*n* = 3). The data is normalized to untreated control. Inhibition of mevalonate pathway in CTL-U251 (**G** and **H**) and TR-U251 (**I** and **J**) cells at 24 and 48 hours following lomitapide treatment (0 μM, 1 μM, or 2 μM) is verified by accumulation of HMGCR. The data are normalized to untreated control. Lomitapide treatment does not lead to accumulation of HMGCR in normal HEK293 cells (**K**). (**L**) Cholesterol uptake by CTL-U251 cells following a 72-hour incubation in serum-free media at various lomitapide concentrations. U-18666A represents positive uptake control. The data are normalized to untreated control. All data is represented as individual measurements with mean ± SD. Two-tailed Student’s *t* tests were used for statistical comparisons between 2 groups, 1-way ANOVA, followed by a post-hoc Tukey’s HSD test, was used for multiple group comparisons. *P < 0.05; **P < 0.01; ***P < 0.001; ****P < 0.0001.

We then sought to clarify the mechanism by which lomitapide sensitizes glioma cells to TMZ treatment. Lomitapide is a cholesterol-lowering agent that acts through inhibition of the microsomal triglyceride transfer protein (MTTP) (13). We hypothesized that the effect of lomitapide on glioma cells was also due to MTTP inhibition. However, lomitapide treatment had no effect on MTTP expression in glioma cells (Figure 4, A–D), suggesting that its mechanism of action in glioblastoma is not by inhibition of MTTP-dependent lipid transfer and assembly. We therefore searched for alternative mechanisms of action of lomitapide in glioma.

Previous work has shown that statins act as antineoplastic agents through inhibition of the mevalonate pathway, an essential biosynthetic step for de novo cholesterol synthesis (17) (Figure 4E). We hypothesized that the effect of lomitapide on glioma cells could similarly be due to mevalonate pathway inhibition. We performed Liquid Chromatography – Mass Spectrometry (LC-MS) on polar metabolite extracts from CTL-U251. We observed significant depletion (85%) of levels of mevalonic acid (metabolite of the reaction catalyzed by the mevalonate pathway rate-limiting enzyme) and 3-hydroxy-3-methylglutaryl-CoA reductase (HMGCR) in CTL-U251 cells treated with 2 μM lomitapide for 72 hours (Figure 4F).

We also examined the effect of lomitapide on mevalonate pathway enzyme expression in glioma. Treatment of CTL-U251 and TR-U251 cells with lomitapide resulted in accumulation of the HMGCR (Figure 4, G–J). Conversely, lomitapide treatment did not result in HMGCR accumulation in HEK293 cells (Figure 4K). These findings confirm our hypothesis that lomitapide inhibits mevalonate pathway by acting on HMGCR.

Notably, lomitapide treatment resulted in a significant decrease in cholesterol uptake by U251 glioma cells cultured in serum-free media (Figure 4L) in a dose-dependent manner. As lomitapide treatment did not result in a change in MTTP expression, we reasoned that decreased cholesterol uptake in glioma cells with lomitapide treatment is due to depletion of cholesterol in the media, secondary to inhibition of intracellular cholesterol synthesis.

**Figure 5:**
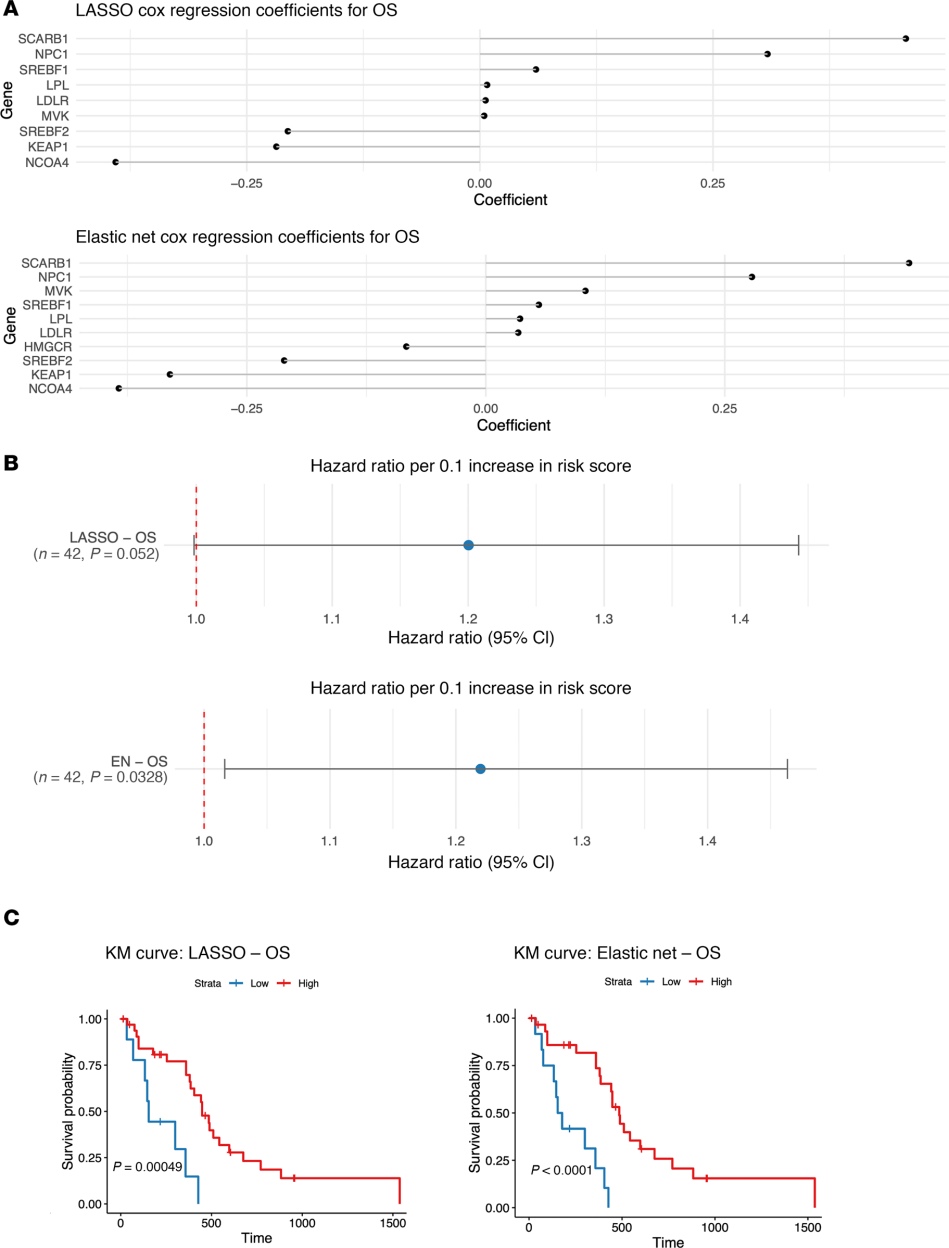
Cholesterol processing gene expression biomarkers are prognostic for OS. (**A**) Coefficient plots for LASSO or Elastic Net regularized Cox models using OS as outcomes and the cholesterol metabolism processing genes from the spatial connectivity score as inputs in newly diagnosed GBM patients from TCGA (*n* = 168) (51). (**B**) LASSO or EN regularized Cox regression hazard ratios for OS per 0.1 increase in the spatial connectivity score. Lines represent 95% CIs. (**C**) Kaplan Meier survival analyses for newly diagnosed GBMs from TCGA (*n* = 168) based on low versus high spatial connectivity score by long rank tests (51).

Previous studies have reported on increased mRNA and protein expression of HMGCR in clinical samples of glioblastoma patients and in glioblastoma cell lines (18). Moreover, HMGCR overexpression has been linked with increased oncogenicity (18). These findings strengthened our rationale for selection of HMGCR as a therapeutic target. Notably, LASSO and Elastic Net (EN) regularized Cox regression models using survival as the endpoint in a cohort of 168 newly diagnosed patients with GBM from the Pan-Cancer Atlas (19) identified cholesterol processing gene expression biomarkers as significantly prognostic for OS (Figure 5, A–C).

**Figure 6:**
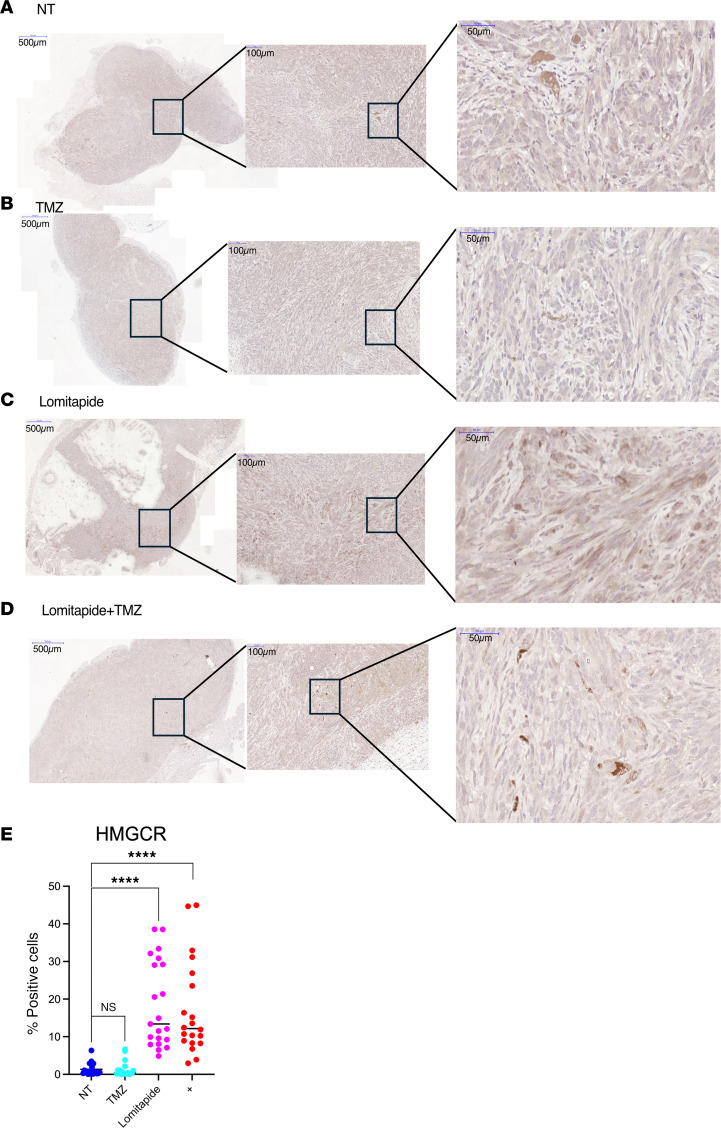
Lomitapide treatment causes accumulation of key mevalonate pathway enzyme HMGCR in vivo. Representative images from IHC analysis of U251 flank tumors stained for HMGCR collected from (**A**) control mice or mice treated with (**B**) TMZ, (**C**) Lomitapide, or a (**D**) combination of Lomitapide and TMZ. The boxes indicate the areas shown at higher magnification. Scale bars: 500 μm (left), 100 μm (middle), 50 μm (right). (**E**) Percentage of cells staining positive for HMGCR as determined through IHC analysis of U251 flank tumors (*n* = 16). All data are represented as individual measurements with mean ± SD. One-way ANOVA, followed by a post hoc Tukey’s HSD test, was used for multiple group comparisons. ****P < 0.0001.

To determine whether the effect of lomitapide treatment also exerts a negative effect on the mevalonate pathway in glioma cells in vivo, we used a heterotopic glioblastoma xenograft model. Mice harboring a U251 flank tumor were divided into 4 treatment groups: (a) control (no treatment); (b) 3 cycles of lomitapide (7.6 mg/kg/day) alone; (c) TMZ (10 mg/kg/day) alone for a 1-week cycle; (d) combination therapy with lomitapide and TMZ. IHC tumor analysis following sacrifice 24 hours after the treatment regimen was completed revealed substantial accumulation of HMGCR in tumors of mice treated with lomitapide alone and mice in the combination group compared with

mice in the control group and mice treated with TMZ alone (Figure 6, A–E).

### Lomitapide primes glioma cells for ferroptosis vulnerability via depletion of CoQ10 and production of ROS.

**Figure 7:**
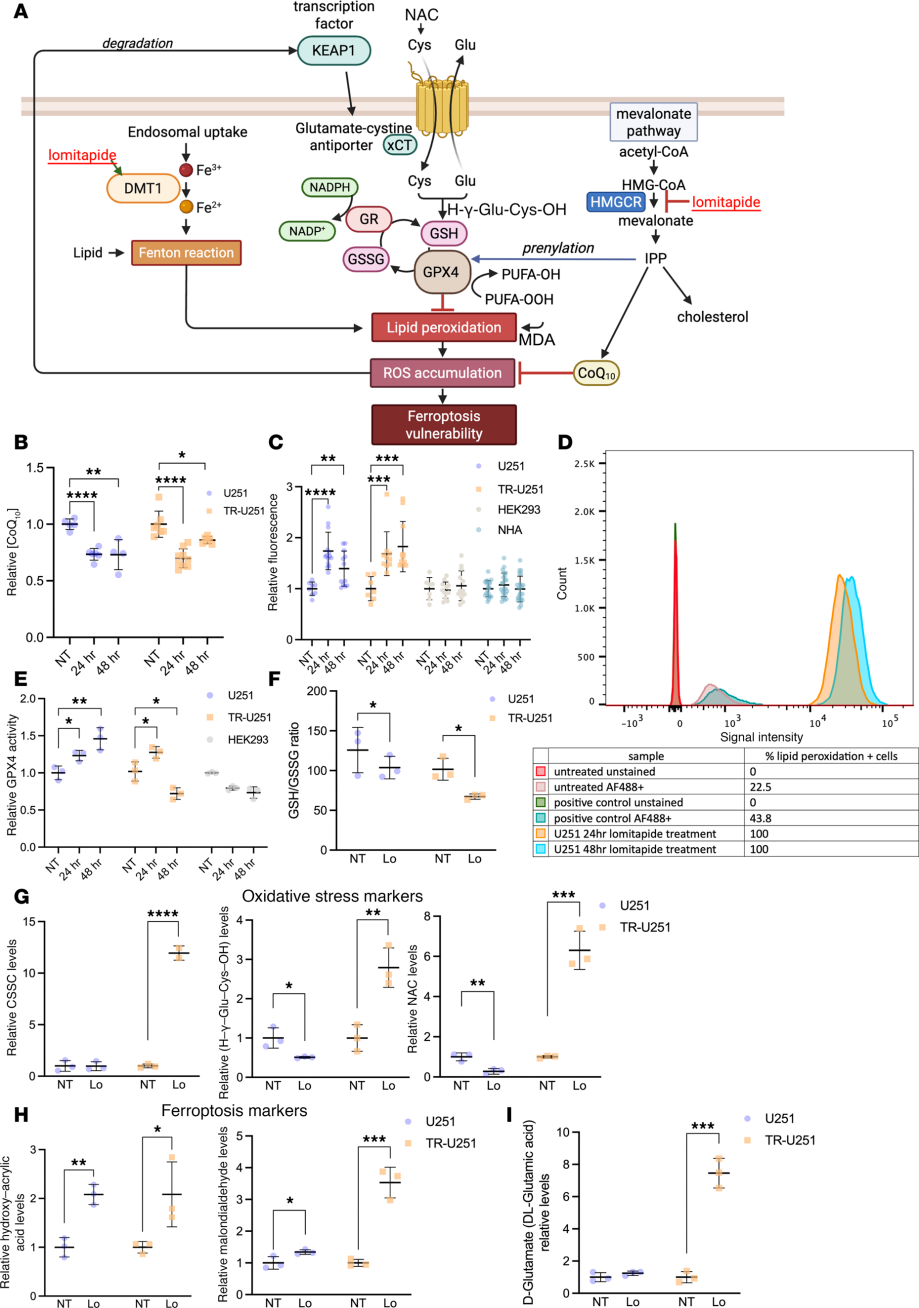
Lomitapide treatment primes glioma cells for ferroptosis. (**A**) Mevalonate pathway inhibition by lomitapide depletes the levels of key antioxidant — CoQ10 — contributing to ROS accumulation. Lomitapide treatment causes overexpression of DMT1, which promotes increase in lipid peroxide levels through fenton reaction. In an adaptive response, GPX4 activity rises to prevent oxidative damage. GPX4 reduces lipid hydroperoxides (PUFA-OOH) to alcohols (PUFA-OH) and then supplements its active site residues through GSH synthesized from cys pumped by system xCT. Expression of glutamate-cystine antiporter is controlled by KEAP1, a critical defense against ferroptosis. Lomitapide primes the cells for ferroptosis. (**B**) Relative CoQ10 concentration in CTL- and TR-U251 cells following 24- and 48-hour lomitapide treatment (2 μM). (**C**) Cellular ROS production in CTL-U251, TR-U251, HEK293, and NHA cells treated with 2 μM lomitapide for 24 and 48 hours. (**D**) Flow cytometry analysis of membrane lipid peroxidation on U251 cells treated with 2 μM lomitapide for 24 and 48 hours. (See Supplemental Figure 3). (**E**) Relative GPX4 activity in cells treated with 2 μM lomitapide for 24 and 48 hours. Untargeted LC-MS results showing GSH/GSSG ratio (**F**), relative concentrations of oxidative stress (**G**); ferroptosis markers (**H**); D-glutamate (**I**) in CTL-U251 and TR-U251 cells treated with 2 μM lomitapide for 72 hours. The data is normalized to untreated control. The data are represented as individual measurements with mean ± SD. One-way ANOVA, followed by post hoc Tukey’s HSD test, used for multiple group comparisons. Cys, cystine; GPX4, Glutathione peroxidase 4; GSH, glutathione; GR, glutathione reductase; xCT, system xCT (glutamate-cystine antiporter); GSSG, glutathione disulfide; NADPH, reduced nicotinamide adenine dinucleotide phosphate; NADP^+^, nicotinamide adenine dinucleotide phosphate; KEAP1, Kelch-like ECH-associated protein 1; DMT1, divalent metal transporter 1; IPP, isopentenyl diphosphate; PUFA-OOH, polyunsaturated fatty acid hydroperoxide; PUFA-OH, polyunsaturated fatty acid alcohol; HMGCR, 3-hydroxy-3-methyl-glutaryl-coenzyme A reductase; MDA, malondialdehyde; HA, hydroxy acrylic acid; CSSC, cysteine glutathione disulfide; H-γ-Glu-Cys-OH, γ-L-Glutamyl-L-cysteine; NAC, N-Acetyl-L-cysteine; NT, no treatment; Lo, 2 μM lomitapide. *P < 0.05; **P < 0.01; ***P < 0.001; ****P < 0.0001.

In addition to its role in cholesterol synthesis, the mevalonate pathway is also critical to the production of the mitochondrial protein, coenzyme Q10 (CoQ10) (20). CoQ10 is a universal multifunctional lipid that plays a fundamental role in cellular ROS homeostasis (20, 21). Due to its high molecular weight and hydrophobicity, systemic transfer of CoQ10 across the BBB is low, leaving cells within the brain reliant on endogenous production (21). We wondered if mevalonate pathway inhibition by lomitapide would deplete CoQ10 levels in highly proliferative glioma cells and cause oxidative stress, thus priming them for chemotherapy-induced cell death (Figure 7A). Indeed, treatment with lomitapide resulted in CoQ10 depletion in both CTL-U251 and TR-U251 cells (Figure 7B). Further, lomitapide treatment resulted in a significant elevation of cellular ROS in CTL-U251 and TR-U251, but not HEK293 or NHA cells (Figure 7C).

ROS accumulation is regarded as one of the hallmarks of ferroptosis, a type of programmed cell death caused by lethal accumulation of lipid peroxides that disintegrates cellular membranes. We assessed the peroxidation of cell membrane lipids, a typical detrimental effect of excess ROS and a hallmark of ferroptosis, by detecting lipid peroxidation–derived protein modifications in fixed cells (22–25). In keeping with the above findings, this oxidative stress parameter was increased by lomitapide treatment in U251 cells (Figure 7D).

Cells are protected from oxidative stress–mediated ferroptosis by the critical antioxidant enzyme glutathione peroxidase 4 (GPX4) (23), a moonlighting protein that reduces lipid hydroperoxides (PUFA-OOH) to alcohols (PUFA-OH) to prevent phospholipid membrane injury (24). GPX4 function is further dependent on the activity of the glutamate-cystine antiporter (system xCT), a key producer of cystine substrate for the synthesis of glutathione, which supplements active site residues of GPX4 enzyme (24). Treatment of CTL-U251 and TR-U251 cells with lomitapide resulted in an initial significant increase in GPX4 enzymatic activity (Figure 7E). However, GPX4 activity was downregulated in TR-U251 cells following prolonged lomitapide treatment, concordant with significant ROS accumulation (Figure 7C).

Glutathione exists primarily in 2 forms: the reduced form (GSH) and the oxidized form (GSSG). GSH is a potent antioxidant that protects cells from oxidative damage by neutralizing ROS, while GSSG is the result of GSH donating electrons to neutralize ROS, becoming oxidized in the process (24). The ratio of GSH to GSSG is a critical indicator of cellular redox status (25). Under normal, healthy conditions, cells maintain a high GSH-to-GSSG ratio, which indicates a reducing environment, necessary for proper cellular function (25). A decrease in GSH-to-GSSG ratio (Figure 7F) suggests that there is an accumulation of oxidative stress or a disruption in the cellular antioxidant defense system, since more GSH is being oxidized to neutralize reactive species. It may serve as an indicator that the cell is at risk of ferroptotic death due to insufficient antioxidant capacity to counteract lipid peroxidation (24).

In CTL-U251 cells, we observed a decrease in relative levels of oxidative markers cysteine glutathione disulfide (CSSC), γ-L-Glutamyl-L-cysteine (H-γ-Glu-Cys-OH), and N-Acetyl-L-cysteine (NAC) following drug treatment (Figure 7G). CSSC is a mixed disulfide that forms between GSH and cysteine, often during oxidative stress (26, 27). A decrease in CSSC may indicate efficient recycling of GSH and cysteine, minimizing disulfide formation and maintaining the pool of free cysteine and GSH needed for antioxidant defense (26, 27). γ-L-Glutamyl-L-cysteine is an intermediate in the synthesis of GSH, formed by the enzyme glutamate-cysteine ligase (26, 27). Its reduction might indicate rapid conversion into GSH, suggesting a heightened demand for GSH synthesis to combat lipid peroxidation, a hallmark of ferroptosis. NAC is a precursor and source of cysteine, indirectly contributing to GSH synthesis (26, 27). A decrease in NAC could imply its utilization to meet the demand for cysteine, supporting the synthesis of GSH to maintain redox homeostasis (26–31). Taken together, these observations suggest active mobilization of antioxidant precursors to counter ferroptotic triggers. Conversely, in TR-U251 cells, we observed significant accumulation of oxidative stress markers CSSC, γ-L-Glutamyl-L-cysteine, and NAC (Figure 7G). Collectively, these changes indicate a weakened capacity to neutralize ROS. The inability to synthesize or utilize GSH effectively compromises the cell’s primary defense against ferroptosis. A disruption in redox homeostasis, failure of antioxidant defenses coupled with reduced GPX4 activity after prolonged lomitapide treatment, and accumulation of oxidative damage suggest that the cell is in a state of ferroptosis or is highly susceptible to it.

A significant increase in malondialdehyde (MDA) and hydroxy acrylic acid (HA) in both CTL-U251 and TR-U251 cells (Figure 7, H and I) indicates elevated oxidative stress and lipid peroxidation, hallmark processes in the context of ferroptosis (29–32). MDA is a byproduct of polyunsaturated fatty acid (PUFA) peroxidation in membranes, catalyzed by iron-dependent processes during oxidative stress (32). MDA is commonly measured as a biomarker for ferroptosis because it directly reflects the extent of lipid peroxidation. HA can be formed as a metabolic byproduct during oxidative stress and altered metabolic fluxes in the tricarboxylic acid (TCA) cycle (31). Elevated HA suggests a cellular shift towards managing oxidative damage or ROS accumulation. HA might reflect secondary oxidative byproducts from amino acid or lipid peroxidation pathways.

**Figure 8:**
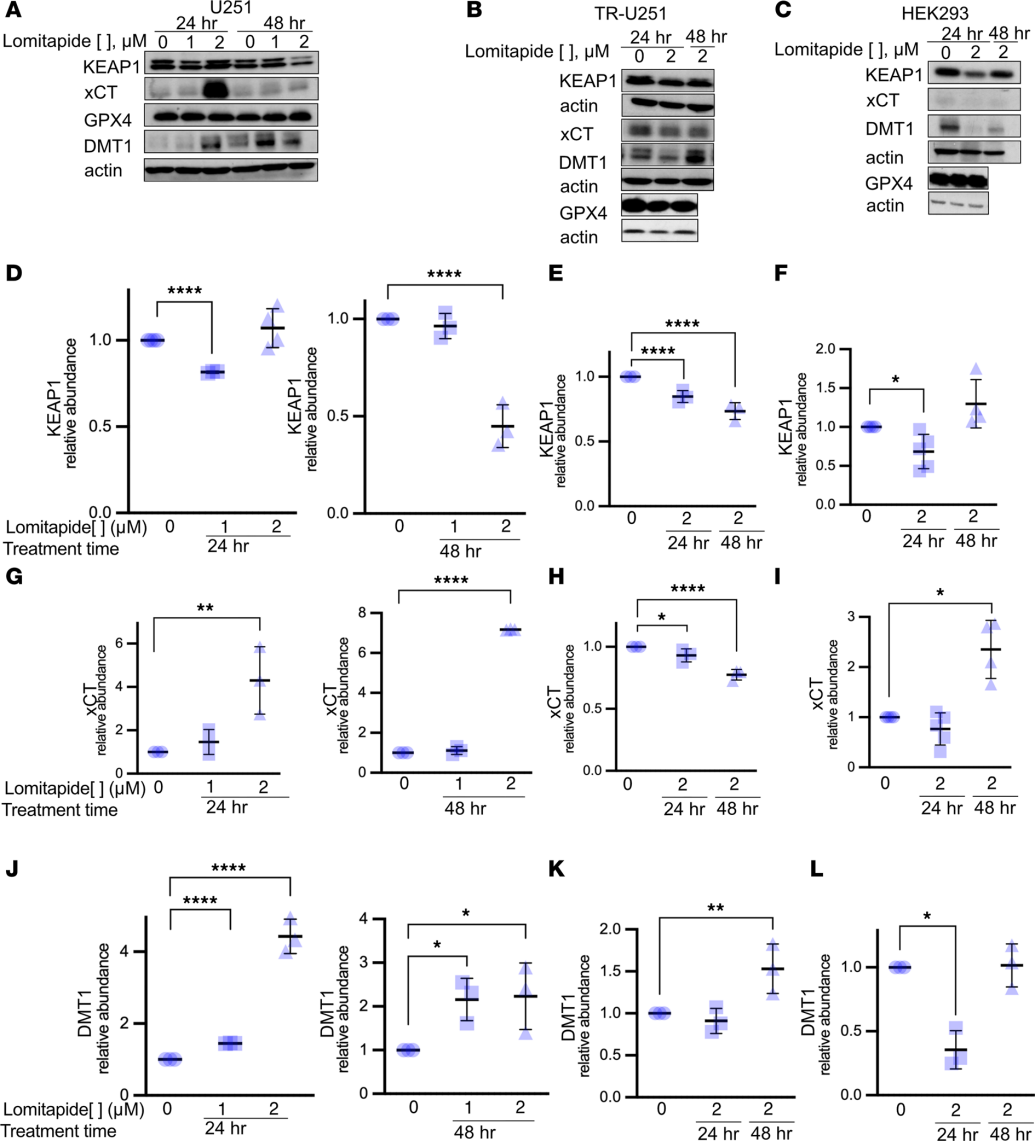
Lomitapide treatment results in expression changes of ferroptotic regulator proteins. (**A**–**L**) Western blot analysis of KEAP1, xCT, GPX4, and DMT1 in CTL-U251 at 24 and 48 hours following 1 and 2 μM lomitapide treatment, TR-U251, and HEK293 cells at 24, 48 hours following 2 μM lomitapide treatment. The data is normalized to untreated control. The data is represented as individual measurements with mean ± SD. One-way ANOVA, followed by a post-hoc Tukey’s HSD test, was used for multiple group comparisons. Cys: cystine, GPX4: Glutathione peroxidase 4, GSH: glutathione, GR: glutathione reductase, xCT: system xCT (glutamate-cystine antiporter), GSSG: glutathione disulfide, NADPH: reduced nicotinamide adenine dinucleotide phosphate, NADP^+^: nicotinamide adenine dinucleotide phosphate, KEAP1: Kelch-like ECH-associated protein 1, DMT1: divalent metal transporter 1, IPP: isopentenyl diphosphate, PUFA-OOH: polyunsaturated fatty acid hydroperoxide, PUFA-OH: polyunsaturated fatty acid alcohol, HMGCR: 3-hydroxy-3-methyl-glutaryl-coenzyme A reductase; MDA, malondialdehyde; HA, hydroxy acrylic acid; CSSC, cysteine glutathione disulfide; H-γ-Glu-Cys-OH, γ-L-Glutamyl-L-cysteine; NAC, N-Acetyl-L-cysteine; N, no treatment; Lo, 2 μM lomitapide. *P < 0.05; **P < 0.01; ***P < 0.001; ****P < 0.0001.

To confirm that ferroptosis is the primary mechanism of cell death, we examined changes in expression of ferroptotic regulator proteins. Lomitapide treatment resulted in significant decrease in the expression of transcription factor responsible for regulation of genes involved in oxidative stress, Kelch-like ECH-associated protein (KEAP1) expression in both CTL-U251 and TR-U251 cells (Figure 8, A–F) (29). We also observed an increase in glutamate-cystine antiporter (system xCT) expression in CTL-U251 cells (Figure 8G). System xCT antiporter imports cystine needed for glutathione synthesis in exchange for exporting glutamate. Glutamate upregulation is a marker of ferroptosis initiation and progression, especially in contexts where system xCT activity is disrupted (24). In TR-U251 cells, system xCT levels were reduced (Figure 8H), causing glutamate to accumulate (Figure 8I) and disrupt glutathione synthesis. This metabolic profile is characteristic of a protective response to oxidative stress, strongly suggesting that the cell is actively resisting ferroptosis.

Iron serves as an indispensable reactive element in ferroptosis, and ferroptosis is accompanied by pronounced intracellular iron accumulation (33). As a proton-coupled metal-ion transport protein, the divalent metal transporter-1 (DMT1) plays a pivotal role in modulating iron homeostasis (33). Increased expression of DMT1 is linked to ferroptosis (34–36). Interestingly, temozolomide-mediated cytotoxicity in glioma has also been found to be dependent on DMT1 (37, 38). Incubation with lomitapide resulted in a substantial increase in expression of DMT1 in CTL-U251 and TR-U251 cells, compared with treatment with vehicle control (Figure 8, J–L). Our findings suggest that treatment of TMZ-resistant cells with lomitapide results in ROS accumulation and sensitizes glioma cells to oxidative stress–mediated ferroptosis.

### Lomitapide delays tumor recurrence and improves survival when combined with TMZ in a glioblastoma xenograft mouse model.

**Figure 9:**
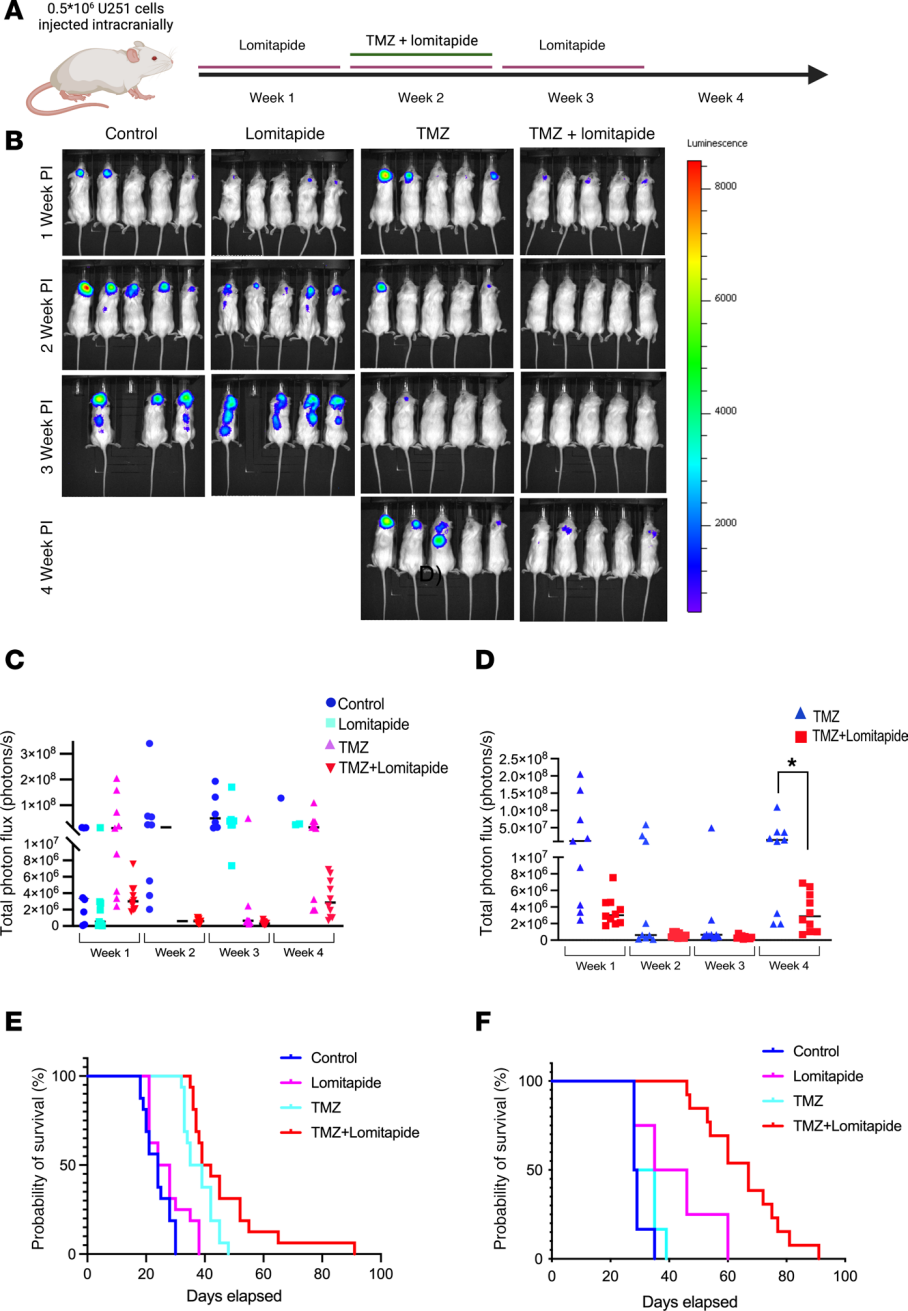
Lomitapide delays tumor recurrence and improves survival when combined with TMZ treatment in a glioblastoma xenograft model. After intracranial inoculation with luciferase-labeled CTL-U251 or TR-U251 cells, mice were randomized into 4 groups. Bioluminescent images were recorded using an IVIS Lumina II Bioluminescence System (PerkinElmer) every 7 days. Total photon flux values were quantified in tumor progression between treatment groups, as described in Sachdeva et al. 2019 (15). (**A**) Schematic showing in vivo experimental design for cells injected intracranially and thereafter treated with lomitapide, TMZ, or concomitant lomitapide and TMZ. (**B**) Bioluminescence imaging of intracranial glioblastoma mouse xenograft visualizing tumor growth of CTL-U251 cells. Empty spaces indicate sacrificed mice at humane endpoints. (**C**) Signal progression of total flux activity comparing tumor growth at 1- to 4-weeks after inoculation. (**D**) Total photon flux of concomitant TMZ and lomitapide-treated and TMZ alone–treated mice 1–4 weeks following transplantation. 2-tailed Student’s *t* test was used for statistical comparison between 2 groups. (**E**) Kaplan–Meier survival curves of mice injected with CTL-U251 in individual cohorts (*n* = 16). *P = 0.0239 for lomitapide + TMZ versus TMZ; **P = 0.0015 for lomitapide + TMZ versus lomitapide; **P = 0.013 for lomitapide + TMZ versus Control. (**F**) Kaplan–Meier survival curves of mice injected with TR-U251 in individual cohorts (*n* = 12). Median survival and statistical significance were determined by log-rank test corrected for multiple comparisons: ****P < 0.0001 for lomitapide + TMZ vs TMZ; *P* < 0.0001 for lomitapide + TMZ vs Control.

Given our in vitro findings that lomitapide enhances glioma cell sensitivity to TMZ through dysregulation of cholesterol and ROS homeostasis, we sought to determine if concurrent lomitapide treatment could enhance the effect of TMZ on tumor growth and survival in vivo. Following intracranial inoculation with luciferase-expressing U251 cells, mice were allocated into 4 treatment groups: (a) control (no treatment); (b) lomitapide (7.6 mg/kg/day) alone; (c) TMZ (10 mg/kg/day) alone; (d) combination therapy with lomitapide and TMZ (Figure 9A). While lomitapide treatment alone demonstrated little antitumor activity or effect on survival at the concentration used for treatment (Figure 9, B and C), the addition of lomitapide to TMZ therapy resulted in a significant reduction in tumor growth (Figure 9, B–D) compared with treatment with TMZ alone (Figure 9, B–E). Tumor recurrence was also substantially delayed in mice receiving combination therapy (Figure 9C), compared with those treated with TMZ alone. Supplementation of TMZ chemotherapy with lomitapide was effective in significantly extending survival in both CTL-U251 and in the resistant TR-U251–inoculated groups, compared with treatment with TMZ alone (Figure 9, E and F).

The safety profile of lomitapide in humans has been established by clinical studies for homozygous familial hypercholesterolemia (HoFH) (12, 39–41). We observed no systemic toxicity in vivo at doses within approved by FDA for treatment of HoFH, highlighting the potential of lomitapide to be translated for clinical application in treatment of GBM.

## Discussion

Our study identifies lomitapide, a lipid-lowering agent that exploits the reliance of glioma cells on de novo cholesterol synthesis, as a chemo-sensitizing agent in glioblastoma. Lomitapide treatment results in CoQ10 depletion and cellular ROS accumulation, which primes glioma cells for ferroptotic death in a DMT1-dependent manner. Further, treatment of with lomitapide enhances the cytotoxic effects of TMZ, delays tumor recurrence, and prolongs survival in a xenograft mouse model of glioblastoma.

Cholesterol is a fundamental component of cellular structure. Notably, the brain contains nearly a quarter of the total cholesterol in the body. To meet their metabolic needs, cancer cells display an increase in de novo cholesterol synthesis and an increase in cholesterol influx or a decrease in cholesterol efflux, to maintain advantageous intracellular cholesterol concentrations (8, 10). Increases in de novo cholesterol biosynthesis are modulated by upregulation of the sterol regulatory element binding protein (SREBP) transcription factors (7). Increases in exogenous cholesterol influx are mediated by an increase in low-density lipoprotein receptor–mediated (LDLR-mediated) endocytosis (8). Lastly, cells establish a surplus of intracellular cholesterol through inhibition of ATP-binding cassette transporter A1–mediated (ABCA1-mediated) efflux (10, 11). Hindrance of these pathways and depletion of extra- or intracellular cholesterol levels via the use of statins and liver x-receptor–agonists (LXR-agonists) have been shown to reduce glioma growth and progression (17).

Cholesterol homeostasis is tightly regulated in the central nervous system to maintain intracellular cholesterol levels at equilibrium (8–11). In diseases such as GBM, this process is disordered. Cancer cells synthesize cholesterol uncontrollably and independent of negative feedback loop regulation (8–11). In fact, glioblastoma cells heavily rely on endogenous cholesterol biosynthesis for survival (11). Therefore, the disruption of cholesterol homeostasis through treatment with lomitapide can be exploited as a vulnerability in TMZ-resistant glioblastoma cells.

The lipid-lowering drug lomitapide first received FDA approval as an adjunct therapy method for patients with HoFH (12). The ability of lomitapide to cross the BBB, decrease exogenous LDL levels, and show only mild adverse effects following standard human doses in current uses, provoked our interest in its potential to be repurposed as a chemotherapeutic agent for glioblastoma. Our findings confirm that, rather than acting on MTTP, lomitapide inhibits the mevalonate pathway in TMZ-resistant glioma cells in a glioma cell–specific manner, resulting in cholesterol and CoQ10 deficiency. Depletion of CoQ10 results in a subsequent increase in ROS burden. Sustained lomitapide treatment also results in decompensation of the GPX4-axis, resulting in further accumulation of lipid peroxides. Treatment-resistant cells are particularly susceptible to disruptions of antioxidant homeostasis and are, therefore, more vulnerable to ferroptosis. Moreover, overexpression of DMT1 promotes increased iron uptake, which contributes to further accumulation of lipid peroxides and ROS through the Fenton reaction (42, 43).

An emerging body of evidence indicates that ferroptosis is crucial for tumor suppression and may reverse drug resistance in canonical therapies (22, 23). Priming cancer cells for ferroptosis has therefore emerged as a promising therapeutic strategy for many malignancies, including gliomas. Ferroptotic death is caused primarily by the peroxidation of phospholipids, which make up the lipid bilayers of cellular membranes (25–29). Specific pharmacological and genetic perturbations can either induce or protect cells from ferroptosis (25–29). Statins have been shown to therapeutically instigate ferroptosis in chemo-resistant cell populations (24).

Yamamoto et al. (2018) have previously shown that glioblastoma cell response to TMZ treatment is modulated by their intracellular cholesterol levels (44). Supporting their findings, cholesterol depletion via glioma cell treatment with lomitapide potentiated the cytotoxic effect of TMZ and resulted in prolonged time to tumor progression and survival in an orthotopic mouse model. Importantly, in our mouse model, lomitapide showed activity at a well-tolerated dose analogous to the clinical adult dose used to treat patients with HoFH. Lomitapide is associated with side effects even in its approved use for HoFH, including liver toxicity and gastrointestinal symptoms. These effects were not investigated in the context of GBM treatment. Lomitapide’s long-term safety profile in patients with GBM, especially when combined with TMZ, requires further investigation.

Through TCGA data analysis, we identified biomarkers predictive of worse OS in patients with GBM. Of note, *NPC1* and *SCARB1* are likely key drivers of poor prognosis. These genes facilitate cholesterol uptake and trafficking, supporting lipid metabolism essential for membrane synthesis and proliferation. Their overexpression may help glioma cells maintain membrane integrity and avoid lipid peroxidation, contributing to increased resistance to ferroptosis-driven cell death. Mevalonate pathway components *KEAP1*, *NCOA4*, *SREBF2*, and *HMGCR* show protective roles, suggesting a potential survival benefit with higher expression, which could relate to metabolic adaptation mechanisms in GBM. These factors might work to enhance endogenous defenses against oxidative stress, including CoQ10 synthesis, which protects against ferroptosis.

In summary, we found that treatment of TMZ-resistant glioma cells with the lipid-lowering drug, lomitapide, resulted in CoQ10 depletion and cellular ROS accumulation, which primes glioma cells for ferroptotic death. Our study identifies lomitapide as a potential therapeutic agent capable of targeting treatment resistance in glioblastoma.

### Limitations of the study and future directions

The accumulation of HMGCR was examined in a subcutaneous (flank) U251 model, which does not accurately reflect the brain microenvironment. However, we have shown that lomitapide has therapeutic benefits in vivo in an orthotopic GBM model.

Most of the available research and clinical focus on lomitapide has been on its effects on cholesterol metabolism in the liver and its efficacy in treating HoFH. Specific studies directly investigating its penetration of the BBB and potential effects within the central nervous system are lacking. However, lomitapide is both is lipophilic and hydrophobic, which enhances its ability to cross lipid-based barriers like the BBB (45). Moreover, a recent study has explored a protective role of lomitapide on ischemic nerve injury (46). Together with our findings in an orthotopic GBM model, we have a strong reason to believe lomitapide can penetrate BBB.

Glioblastoma is an extremely heterogeneous disease, a factor that poses a substantial challenge in developing effective therapeutics. Recent single-cell studies have shown that a single GBM tumor can harbor multiple subtypes, complicating therapeutic targeting (47). GBM cells are highly plastic and can acquire different molecular features depending on the microenvironment or therapeutic pressure. This study does not address how lomitapide might influence this plasticity, which is crucial for developing long-term effective therapies. Moreover, a substantial portion of the functional experiments were performed using long-term cultured glioblastoma cell lines such as U251, which do not faithfully recapitulate the molecular features of clinical glioblastoma. To enhance the clinical relevance and assess how lomitapide might influence or interact with the immune response in glioblastoma, future studies should focus on validating the efficacy of lomitapide across other subtypes of GBM using patient-derived xenograft models in humanized mouse models.

Through LASSO and EN regression models we identified several biomarkers predictive of OS in the GBM patient population. Future studies should focus on deciphering the specific functional and mechanistic contributions of those players on a larger scale.

This study provides evidence for ferroptosis being the primary cell death mechanism of lomitapide-treated cells but doesn’t delve into alternative mechanisms or compensatory pathways that might counteract ferroptosis induction. Future studies should investigate auxiliary mechanisms of cell death and monitor ferroptosis markers dynamically in live cells.

## Methods

### Sex as a biological variable

Our study examined male and female animals, and similar findings are reported for both sexes.

### Study design

#### Research objectives.

The primary objective of this study is to evaluate the potential therapeutic effects of lomitapide in combination with temozolomide on glioblastoma cell lines and xenograft models. Specifically, this study aims to assess cell viability, drug synergy, and the underlying molecular mechanisms responsible for the effects of these drugs, including lipid peroxidation and glutathione peroxidase activity. We hypothesize that lomitapide may enhance the efficacy of TMZ in glioblastoma treatment, potentially through the induction of oxidative stress and lipid peroxidation.

#### Research subjects and units of investigation.

The cell lines used include immortalized human glioblastoma cell lines (U251, TR-U251), human neural stem cells (NHAs), and HEK293 cells. Glioblastoma Stem Cells (GSCs) used include G811, G818, and GliNS1 GSC lines. the animal models used include NOD SCID gamma (NSG) mice aged 6–10 weeks, both male and female, for flank and intracranial xenograft studies. The sample size was determined based on prior experiments and statistical power calculations to ensure adequate power to detect meaningful effects. 

### Experimental models and participant details

#### Cell lines.

The immortalized human glioblastoma cell line U251, immortalized NHAs (48),and the human embryonic kidney cell line HEK293 were purchased from the American Type Culture Collection (ATCC). Both lines were grown as adherent cells and cultured in DMEM (Cat#319-050-CL, Wisent, ST-BRUNO, Quebec Canada) supplemented with 10% FBS (Cat#920-040, Wisent) and 1% penicillin/streptomycin (P/S) (Cat# 450-200-EL, Wisent).

The GSC lines G811, G818 (a gift from Frederick Lang, Department of Neurosurgery, MD Anderson Cancer Center [Houston, Texas, US]), and GliNS1 (a gift from Peter Dirks, Hospital for Sick Children (Toronto, Ontario, Canada)) were cultured as neurospheres in DMEM F-12 (Cat# 319-075-CL, Wisent) supplemented with 2 mmol/L L-glutamine (Cat# 25030081, Thermo Fisher Scientific Inc), 1× antibiotic/antimycotic (Cat# 15240062, Thermo Fisher Scientific Inc), 2% B27 supplement (Cat# 17504044, Thermo Fisher Scientific Inc), 20 ng/mL human epidermal growth factor (hEGF, Cat#9644, Sigma), and 20 ng/mL human basic fibroblast growth factor (hFGF, Cat# F5542, Sigma).

All cell lines were maintained at 37°C and 5% CO_2_.

#### Chemotherapeutic agents.

Temozolomide (Sigma, T2577) and lomitapide (Sigma, SML1385) were reconstituted in DMSO prior to use.

#### High-throughput screening.

HTS was made possible via the S.M.A.R.T. Laboratory for High-Throughput Screening Programs’ research facility, part of the Sinai Health System. SelleckChem’s FDA-approved drug library (Catalog #L1300) was used for ease of drug repurposing. CTL- and TR-U251 cell lines were seeded into 384-well plates (500 cells/well) on Day 0. The approximate 900 drug agents, alongside negative (DMSO, final concentration 1%) and positive (TMZ) controls, were pinned the following day at 0.5 and 4.0 μM concentrations using a Biomek FX (Beckman Coulter). Plates were incubated in DMEM 10% FBS at 37°C and 5% CO_2_. Cell confluency was monitored on Day 3. Population growth and cell viability were determined on Day 4 by means of AlamarBlue Cell Viability Assay (Thermo Fisher Scientific), per the manufacturer’s instructions. Additional readout from this experiment included “hit” status, B-score (B-score refers to a statistical method used to normalize data from HTS experiments, particularly in identifying potential drug candidates, by correcting for systematic variations across a plate, such as row and column effects, allowing for a more accurate analysis of compound activity. B-score is considered a robust normalization procedure that utilizes the Tukey median polish algorithm to remove these plate-related biases (49), ability to cross the BBB, previously unreported use in the CNS, and target pathway). These data were then used to select 8 prospective drug candidates for further analysis.

For validation, U251 cell lines were seeded into 384-well plates as described above. The eight-drug leads were pinned in quadruplicate the following day using serially increasing concentrations ranging from 0.039 to 20.0 μM. After 72 hours, AlamarBlue was again used to assess cell viability. The readout for this experiment included dose-response figures with corresponding IC_85_ and IC_50_ values.

#### Cell viability assays.

For more stringent testing on potential drug leads, adherent (1,000 cells/well) and sphere (2,000 cells/well) cells were seeded in 96-well plates. Cell numbers were determined using a Vi-CELL XR cell counter (Beckman Coulter). The following day, cells were treated with drug(s) in 8 × replicates and maintained for 72 hours in culture. CellTiter-Blue Reagent (Promega) was then added to cells (20 μL/well). After a 2-hour incubation period, cell growth and viability were measured fluorometrically (560 E_x_/590 E_m_) using a VersaMax Microplate reader (Molecular Devices).

#### Lomitapide in vitro treatment.

2 × 10^6^ cells were seeded in T25 flasks in DMEM (1% FBS, 1% P/S media). Cells were treated with 0 μM, 1 μM, or 2 μM lomitapide diluted in DMEM (1% FBS, 1% P/S).

#### Drug synergy.

We used SynergyFinder (16) to evaluate drug combination synergy using 4 models. (a) A ZIP (Zero Interaction Potency) score greater than 0 typically suggests that the combination of the 2 drugs is synergistic rather than additive or antagonistic. (b) A Bliss score greater than 1 indicates that the combination is more effective than the individual drugs would be expected to be based on their independent effects. (c) A Loewe synergy score greater than 0 indicates synergy, where the drugs work together more effectively than expected. (d) A HSA (Highest Single Agent) synergy score measures how much more effective the combination is compared to the most potent drug alone. A value greater than 0 suggests synergy. For general reporting, the Bliss and Loewe synergy scores are usually the most informative and are typically favored for reporting in drug combination studies, especially when showing synergy.

#### Colony-forming assay.

A total of 3 **×** 10^4^ U251 and TR-U251 cells were plated as described above and treated with 100 μM temozolomide, 2 μM lomitapide, or the combination of both drugs for 7 days, after which the cells were washed with PBS and cultured in fresh media for 7 days. The cells were then fixed in 10% formalin for 15 minutes and incubated in 0.01% crystal violet dye for 1 hour. Thereafter, the wells were washed with water 3 times. The plates were scanned. Three out of 5 representative images per condition are shown. ImageJ was used to quantify the number of colonies and measure colony area.

#### Sphere formation assay.

Cells pretreated for 72 hours were dissociated into single-cell suspensions using accutase (Thermo Fisher, Cat # 00-4555-56). Cell pellets were washed with 1 × HBSS (InVitrogen/Gibco 14025-092) and resuspended in staining medium containing 1 μg/mL of propidium iodide (Molecular Probes, P1304) to eliminate dead cells. Single live cells were sorted into 96-well plates containing neurosphere culture media using Aria Fusion A or Sony MA900. To calculate the forming efficiency, spheres were scored 4 weeks after plating, and images were obtained using the EVOS FL Auto Imaging System (Thermo Fisher Scientific). The forming efficiency (% spheres formed) = scored sphere number / total plating cells.

#### Cholesterol uptake assay.

A Cholesterol Uptake Assay Kit (Abcam) was used to measure the amount of cholesterol consumed by cultured cells. Briefly, CTL-U251 cells were plated at 1,000 cells/well in DMEM 10% FBS plus P/S media in a standard 96-well plate. After 24 hours, the serum-supplemented media was removed and replaced with serum-free media. Next, wells were subjected to 1 of 3 treatment groups: (a) U-18666A, a positive control reagent included in the assay kit; (b) no treatment; (c) lomitapide at 1.0, 1.5, 2.0, and 2.5 μM concentrations. Lastly, the fluorescent-tagged cholesterol probe was added to each well (2 μL/well) to obtain a final concentration of 20 μg cholesterol/mL of culture medium. After a 72-hour incubation period, plates were analyzed on a plate reader at the excitation and emission wavelengths of the fluorescent probe (485 nm and 535 nm, respectively).

#### Human CoQ10 ELISA.

Cells pretreated with lomitapide were collected with PBS and homogenized with rotor stator. Samples were prepared at 0.1 × 10^6^ dilution with 1 × sample diluent. Samples were treated with 1 × HRP conjugate, per the manufacturer’s instructions, and incubated for 40 minutes at 37°C. Following 5 washes with the supplied Wash Buffer, the samples were incubated with TMB substrate for 20 minutes at 37°C and protected from light. Stop solution was added to each sample and the optical density was measured at 450 nm using a microplate reader.

#### GPX4 enzyme activity assay.

GPX4 enzyme activity was measured in lomitapide-treated and control CTL-U251, TR-U251, and HEK293 cell lines using Glutathione Peroxidase Assay Kit (Colorimetric, Abcam #102530) according to manufacturer’s cell lysate protocol. Adherent cells were grown in standard cell culture media at 2 × 10^6^ cells and treated with 2 μM lomitapide for 24 or 48 hours. On the day of the experiment, the cells were collected with 1 × assay buffer and homogenized with rotor stator. GPX4 reaction mix was added to the samples and the samples were incubated for 15 minutes at room temperature to deplete all GSSG. The OD was measured at 340 nm on a microplate reader prior to and following the glutathione peroxidase reaction, which was initiated with cumene hydroperoxide solution. Active Glutathione Peroxidase/Glutathione Peroxidase was used as a positive control. The unit of GPX4 activity was defined as the amount of enzyme that causes the oxidation of 1.0 μmol of NADPH to NADP^+^ under the assay kit condition per minute at 25°C and normalized to the control.

#### Lipid peroxidation assay.

Cells were plated and incubated in complete growth medium at 37**°**C. For lomitapide treatment, the cells were pretreated with 2 μM lomitapide for 24 and 48 hours. The cells were then treated with vehicle (DMSO) or positive control 100 μM cumene hydroperoxide (CH) and then immediately fed with 50 M Click-it LAA and incubated for 2 hours at 37**°**C. The cells were then fixed with 4% formaldehyde for 15 minutes at RT. The cells were washed 3 times with PBS and permeabilized with 0.05% Triton X-100 for 10 minutes. The cells were blocked with 1% BSA for 30 minutes. The cells were washed and click reaction performed with 5 M Alexa Fluor 488 azide for 30 minutes and then washed 1 time with 1% BSA and 2 times with HBSS. Lipid peroxidation was quantified by flow cytometry on BD Fortessa UVBGR. Signal intensity on B515 flow channel was analyzed using FlowJo 10.10.0 software. Percentage of lipid peroxidation–positive cells was reported. The gating strategy is reported in Supplemental Data.

#### IHC.

HMGCR enzyme expression studies were performed on samples of heterotopic glioblastoma xenografts. Paraffin-embedded blocks were cut into 5-μm sections and dewaxed in xylene, followed by rehydration in a standard alcohol series (90%, 70%, and 50%). Antigen retrieval was achieved by 20 minutes of pressure cooking in citrate buffer (pH 6.0), followed by blocking for 10 minutes with Universal Blocking Buffer (Cat# X0909, Dako). The slides were then incubated with antibody for HMGCR (Cat# ab242315, Abcam) overnight at 4°C. Detection used biotinylated secondary antibodies for 1 hour, the ABC Reagent Kit (Vector Labs), and DAB chromogen (Vector Labs). Slides were washed with PBS 3 times after each step. Sections were then dehydrated using increasing concentrations of ethanol (50%, 70%, and 90%), followed by a brief washing in xylene. Finally, the slides were mounted in Permount (Thermo Fisher Scientific). Immunoexpression of proteins of interest was quantified with Halo Image Analysis software v4.0 (Indica Labs) to calculate the percentage of positive cells over a designated tissue area.

#### Western blotting.

Protein extraction was performed at different time points (24 or 48 hours of treatment). Fifty μg of protein sample per treatment condition was loaded to assess protein expression levels. Membranes were blotted with antibodies for HMGCR (Cat# ab242315, Abcam), DMT1/SLC11A2 (D3V8G, Cat# 15083, Cell Signaling Technology), GPX4 (Cat# 52455, Cell Signaling Technology), KEAP1 (D6B12, Cat# 8047, Cell Signaling Technology), xCT/SLC7A11 (D2M7A, Cat# 12691, Cell Signaling Technology), or β-Actin (Cat# 4970, Cell Signaling Technology). Bound antibodies were detected with horseradish peroxidase-linked anti-mouse or anti-rabbit IgG (Cell Signaling Technology), followed by ECL (PerkinElmer). See also the Key Resources Table of the Supplemental Materials. All samples were run in triplicate.

#### Cellular ROS assay.

We used the DCFDA - Cellular ROS Assay Kit / Reactive Oxygen Species Assay Kit (Abcam #113851) uses the cell permeant reagent 2’,7’ –dichlorofluorescin diacetate (DCFDA, also known as H2DCFDA, DCFH-DA, and DCFH) and semiquantitatively assessed ROS in live cell samples. Hydroxyl, peroxyl, and other ROS were measured in lomitapide-treated and control CTL-U251, TR-U251, NHA, and HEK293 cell lines according to manufacturer’s adherent cells protocol for microplate assay. Adherent cells were grown in standard cell culture media at 1,000 cells/well and treated with 2 μM lomitapide for 24 or 48 hours. On the day of the experiment, the cells were washed with 1 × buffer, stained with the diluted DCFDA solution (100 μL/well), and incubated at 37°C for 45 minutes in the dark. The plate was immediately measured on a fluorescence plate reader at Ex/Em = 485/535 nm in end point mode in presence of 1 × buffer. TBHP was used as positive control.

#### Quantification of metabolite concentrations.

CTL-U251 and TR-U251 cells (10 × 10^6^ cells/plate) were seeded in 150 × 25 mm cell culture dishes (Falcon) in DMEM with 10% FBS and 1% P/S media. Cells were grown for 72 hours in 2 μM lomitapide. Metabolites were extracted using –20°C extraction buffer (40% methanol, 40% acetonitrile, 20% H2O, 0.1 mM Formic Acid). The samples were prepared for LC-MS analysis as described by Rabinowitz and Kimball (50). The extracts were dried using a nonheat vacuum dryer (Thermo Fisher Scientific, Savant RVT5105 Refrigerated Vapor Trap and Savant SPD121P SpeedVac Concentrator). The samples were submitted to the BioZone at the University of Toronto for LC-MS analysis using an Exactive orbitrap mass spectrometer equipped with a heated electrospray ionization source and either a Hypersil Gold C18 column (50 mm × 2.1 mm, 1.9 μm particle size; Thermo Fisher Scientific) or an Atlantis Premier BEH Z-HILIC column (100mm × 2.1mm, 1.7 μm; Waters). See Supplemental methods for full details. The samples were run in triplicate.

#### LASSO and EN regression models for cholesterol processing genes.

Development and validation of risk scores were performed using RNA-seq and clinical data from TCGA pan-cancer atlas (19). Samples were filtered for newly diagnosed GBMs. The resulting cohort was split into 75% training and 25% testing set. Gene expression values were log_2_ transformed and used to train LASSO and EN regularized Cox regression models (with 10-fold cross validation) to predict OS with the concordance (c) index for each target endpoint, as described by Raleigh et al. (51). To measure model performance, we employed c-index in univariable Cox proportional hazards models in the testing set using the survival (v3.7-0) package in R, as described by Raleigh et al. (51). We divided risk scores using the maximally selected rank statistic into 2 risk groups — low or high connectivity, using the surv_cutpoint function from the survminer (v0.5.0) package in R and employed the Kaplan Meier method and log-rank test for risk stratification.

### Animal models

#### Flank xenograft mouse models.

NOD SCID γ (NSG) mice aged 6–10 weeks old (both sexes) were purchased from the Jackson Laboratory. Luciferase-tagged CTL-U251 cells (2 × 10^6^) were resuspended in 100 mL of 1:1 in media: Matrigel (catalog no. 354234, Corning) and injected subcutaneously in the flank. Tumor progression was tracked by bioluminescence imaging. Seven days after injection, mice were randomized into 4 treatment groups (*n* = 4/group): temozolomide (10 mg/kg/day for 1 cycle of 5 days), lomitapide (7.6 mg/kg/day for 3 cycles of 5 days), concomitant temozolomide and lomitapide (10mg/kg TMZ for 1 cycle of 5 days + 7.6 mg/kg lomitapide for 3 cycles of 5 days administered as neoadjuvant and adjuvant to TMZ), or vehicle control. All drugs were reconstituted in DMSO and further diluted in water prior to use. TMZ treatment was administered intraperitoneally, and lomitapide-treatment was administered by oral gavage. Mice were sacrificed 24 hours after treatment completion. The tumors were collected, sectioned, and examined by IHC.

#### Intracranial xenograft mouse models.

NSG mice aged 6–10 weeks old (both sexes) were purchased from the Jackson Laboratory. Animal studies were carried out at The Centre for Phenogenomics (TCP) in compliance with Animal Care Committee (ACC) and Canadian Council on Animal Care (CCAC) policies. Luciferase-tagged CTL-U251 and TR-U251 cell line (0.5 × 10^6^) were used for stereotaxic intracranial implantation into NSG mice. One week after inoculation, mice underwent bioluminescent imaging and were randomized into treatment groups: vehicle control, TMZ (10 mg/kg), lomitapide (7.6 mg/kg), or concomitant treatment (10mg/kg TMZ + 7.6 mg/kg lomitapide). All drugs were reconstituted in DMSO and further diluted in water prior to use. TMZ treatment was administered intraperitoneally, and lomitapide treatment was administered by oral gavage. Temozolomide treatment method began 1 week after cell inoculation and was repeated daily for 5 days. Lomitapide treatment method began 1 day after cell inoculation and was repeated daily for 3 weeks. Tumor progression was monitored weekly using an IVIS Lumina Bioluminescence System (PerkinElmer). Mice were observed regularly and euthanized upon notice of deteriorating health (weight loss, weakness, doming of the head, hunched vertebrae, etc.). Tissue was obtained for histological analysis.

#### Bioluminescent imaging.

In vivo tumor progression was monitored weekly using bioluminescent imaging (BLI). First, mice were anesthetized using 2% isoflurane, then injected intraperitoneally with 0.15 mL of luciferin (15 mg/mL). A 3-minute wait period proceeded injection to allow adequate time for the chemical to reach the target tumor cells. Next, an IVIS Lumina System (PerkinElmer) was used to image the mice. Results were analyzed using Living Image 4.3.1 software and total flux values were calculated.

#### Dose translation.

As suggested by the FDA, body surface area (BSA) normalization was used to determine human to mouse dose translation for in vivo studies (52) using the following formula:


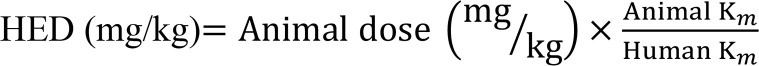


where HED is the human equivalent dose and K*_m_* is the factor used to convert body weight into surface area. The K*_m_* factor for a mouse is 3 and that of a human is 37.

### Statistics

All experiments were performed with 3–10 biological replicates. Technical replicates were also included in each experiment. Graphed data are expressed as individual values with mean ± SD, unless otherwise stated. For direct comparisons, significance was determined by the unpaired (independent groups) or paired (repeated measures or matched data) 2-tailed Student’s *t* test with Bonferroni correction. One-way ANOVA, followed by a post hoc Tukey’s HSD test, was used for multiple group comparisons in normally distributed homoscedastic data. GraphPad Prism 10 software was used to analyze results. *P* < 0.05 was considered statistically significant.

### Study approval

All procedures involving animals were reviewed and approved by the Institutional Animal Care Committee at Toronto Center for Phenogenomics, Toronto, Canada. St. Michael’s Hospital REB #19-311. The Hospital for Sick Children REB #1000024587.

### Data availability

All data are available in the main text or the Supplemental Materials. Supporting data values are provided in the Supporting Data Values file in the Supplemental Materials.

This study did not generate new unique reagents. Any additional information required to reanalyze the data reported in this paper is available from the lead contact upon request.

## Author contributions

Conception and design: AI, MW, TMW, and SD. Development of methodology: AI, MW, TMW, and SD. Acquisition of data: AI, MW, TMW, SD, KGZ, RF, and ET. Analysis and interpretation of data: AI, MW, TMW, RF, and SD. Writing, review, and/or revision of the manuscript: AI, MW, TMW, and SD. Administrative, technical, or material support (i.e., reporting or organizing data, constructing databases): AI, MW, and TMW. Study supervision: MW and SD.

## Supplementary Material

Supplemental data

Unedited blot and gel images

Supporting data values

## Version 1 07/23/2025

In-Press Preview

## Version 2 09/09/2025

Electronic publication
