# Supplementary material for: Lomitapide enhances cytotoxic effects of temozolomide in chemotherapy-resistant glioblastoma
Source: JCI Insight. 2025 Jul 22;10(17):e186703. doi: 10.1172/jci.insight.186703 (PMC12487684; doi:10.1172/jci.insight.186703)
Supplement: Supplemental data [file jciinsight-10-186703-s179.pdf]

Supplementary Figure S1. Chemical structure of lomitapide (source: https://www.selleckchem.com/products/lomitapide.html).

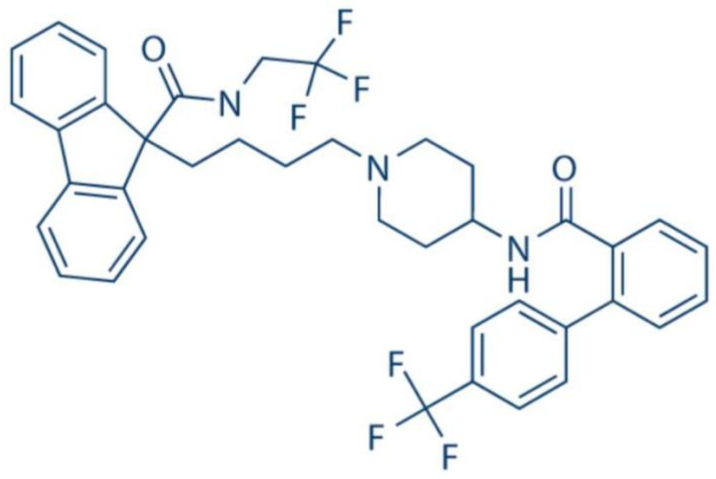

Supplementary Figure S2. Bliss, HSA, Loewe, and ZIP synergy maps for **(A)** CTL-U251 and **(B)** TR-U251 cells.

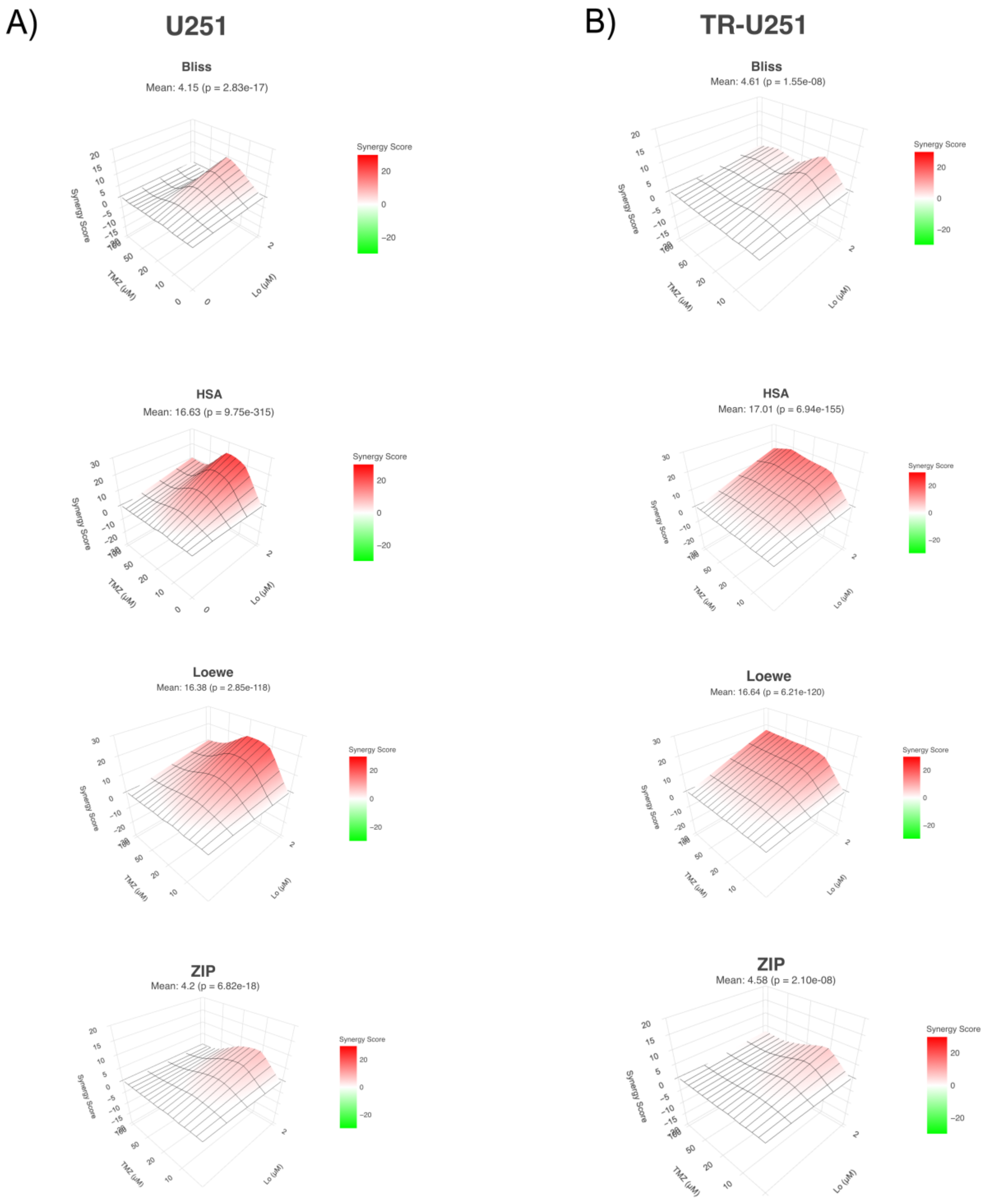

Supplementary Figure S3. Gating strategy for flow cytometry analysis of CTL-U251 cells. Viable cells were selected from a forward scatter-area vs side scatter-area dot plot, and single cells were subsequently selected in a forward scatter-area vs forward scatter-area dot plot. Then, lipid peroxidation-positive cells were selected by AF488 signal. **(A)** untreated unstained control; **(B)** untreated AF488+; **(C)** positive control unstained; **(D)** CTL-U251 24-hr lomitapide (2μM) treatment; **(E)** CTL-U251 48-hr lomitapide (2μM) treatment; **(F)** positive control AF488+.

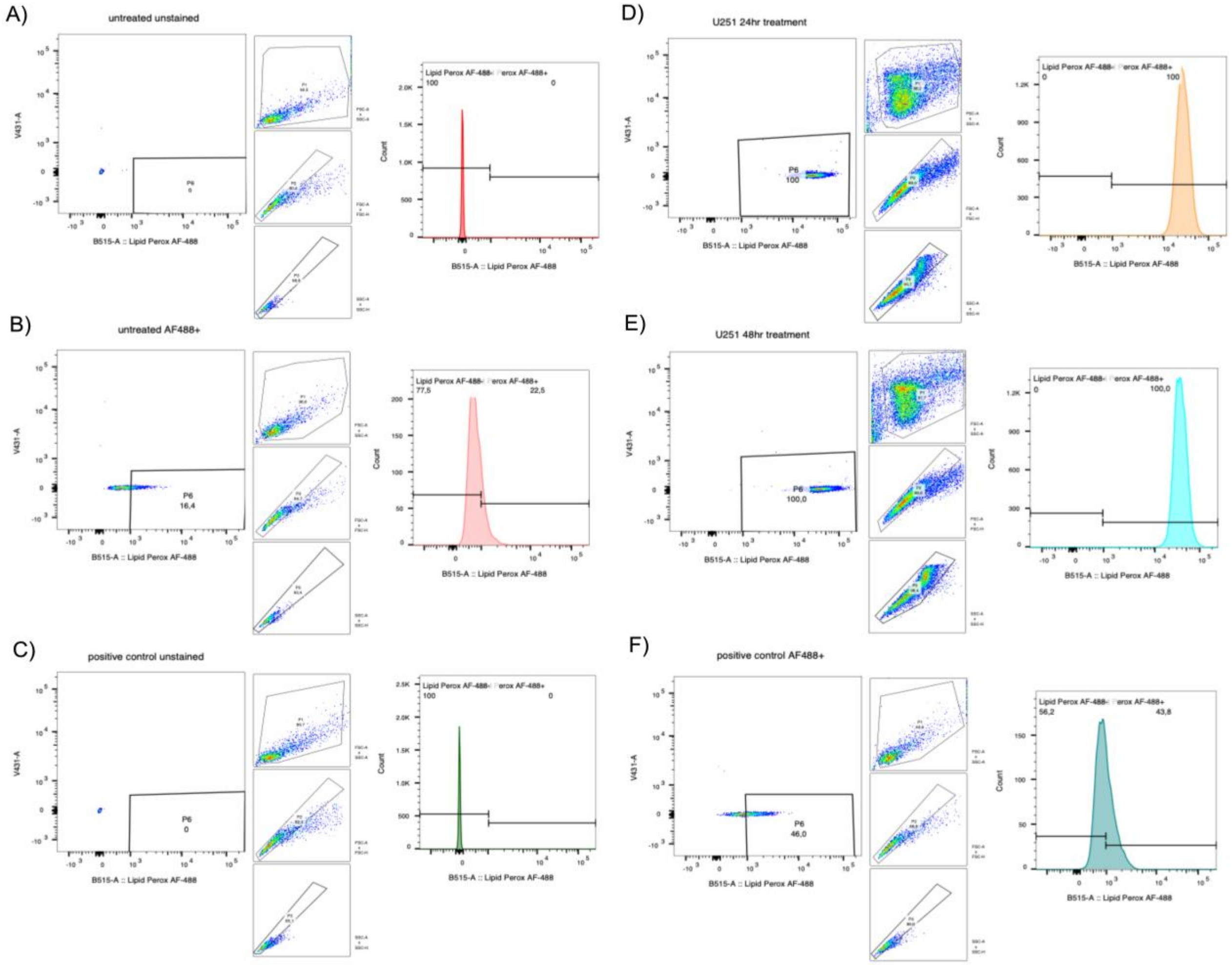

Supplemental Table S1. Lead candidates identified through the high-throughput drug screen of FDA-approved agents capable of crossing the blood-brain barrier.

drug name	drug type	mechanism of action	ability to cross the BBB	reference
pralatrexate	folate analog metabolic inhibitor antineoplastic agent	Competitively inhibits dihydrofolate reductase (DHFR) and polyglutamylation by the enzyme folylpolyglutamyl synthetase (FPGS) in cells expressing reduced folate carrier type 1 (RFC-1). results in the inhibition of RNA synthesis, DNA replication and cancer growth and apoptosis	+	National Center for Biotechnology Information. PubChem Compound Summary for CID 148121, Pralatrexate. https://pubchem.ncbi.nlm.nih.gov/compound/Pralatrexate. Accessed Oct. 3, 2023.
dronedarone	antiarrhythmic agent	Multichannel blocker (Na, K, and Ca ion channels and inhibiting β-adrenergic receptors) that controls rhythm and rate in atrial fibrillation	+	National Center for Biotechnology Information. PubChem Compound Summary for CID 208898, Dronedarone. https://pubchem.ncbi.nlm.nih.gov/compound/Dronedarone. Accessed Oct. 3, 2023.
lomitapide	lipid-lowering agent	microsomal triglyceride transfer protein inhibitor used to lower cholesterol associated with homozygous familial hypercholesterolemia (HoFH), reducing risk of cardiovascular events such as myocardial infarction and stroke	+	National Center for Biotechnology Information. PubChem Compound Summary for CID 9853053, Lomitapide. https://pubchem.ncbi.nlm.nih.gov/compound/Lomitapide. Accessed Oct. 3, 2023.
ethacridine lactate monohydrate	anti-infective agent	DNA intercalating agent, indirectly inhibits poly(ADP-ribosyl) glycohydrolase (PARG) by forming a complex with PAR and blocking the binding of PARG to substrate	+	National Center for Biotechnology Information. PubChem Compound Summary for CID 165457, Ethacridine lactate monohydrate. https://pubchem.ncbi.nlm.nih.gov/compound/Ethacridine-lactate-monohydrate. Accessed Oct. 3, 2023.
delanzomib	P2 threonine boronic acid inhibitor	Chymotrypsin-like activity of the proteasome, with potential antineoplastic activity. Represses the proteasomal degradation of a variety of proteins, including IκBα resulting in the cytoplasmic sequestration of the transcription factor NF-κB; inhibition of NF-κB nuclear translocation and transcriptional up-regulation of a variety of cell growth-promoting factors; and apoptotic cell death in susceptible tumor cell populations	+	National Center for Biotechnology Information. PubChem Compound Summary for CID 24800541, Delanzomib. https://pubchem.ncbi.nlm.nih.gov/compound/Delanzomib. Accessed Oct. 3, 2023.
omipalisib	small-molecule pyridylsulfonamide inhibitor of phosphatidylinositol 3-kinase (PI3K)	Binds to and inhibits PI3K in the PI3K/mTOR signaling pathway, which may trigger the translocation of cytosolic Bax (member of the proapoptotic Bcl2 family of proteins) to the mitochondrial outer membrane, increasing mitochondrial membrane permeability and inducing apoptotic cell death	+	National Center for Biotechnology Information. PubChem Compound Summary for CID 25167777, Omipalisib. https://pubchem.ncbi.nlm.nih.gov/compound/Omipalisib. Accessed Oct. 3, 2023.
tivantinib	small molecule inhibitor of c-Met	Binds to the c-Met protein and disrupts c-Met signal transduction pathways, which may induce cell death in tumor cells overexpressing or expressing constitutively activated c-Met protein	+	National Center for Biotechnology Information. PubChem Compound Summary for CID 11494412, Tivantinib. https://pubchem.ncbi.nlm.nih.gov/compound/Tivantinib. Accessed Oct. 3, 2023.

Supplementary Methods

HILIC Chromatography
UHPLC: Thermo Scientific Ultimate 3000
Column: Waters Atlantis Premier BEH Z-HILIC 1.7 µm, 2.1 x 100 mm (equipped with guard column)
Column Temperature: 40degC
Autosampler temperature: 5degC
Injection Volume: 10uL
Solvent A: 15mM Ammonium Bicarbonate pH 9 in water
Solvent B: 15mM Ammonium Bicarbonate pH 9 in 90% water, 10% H2O
Flow Rate: 500uL/min
Gradient: 0min @ 90%B, 0-5min gradient to 65%B, 5-6min @ 65%B. 6-7min gradient to 90%B, 7-13min @ 90%B

Mass Spectrometer Settings
Mass Spectrometer: Thermo Scientific Q Exactive
Source: HESI II
Spray Voltage: 3.5kV
Capillary Temperature: 320degeC
Sheath Gas: 40
Aux Gas: 20
Spare Gas: 5
S-Lens RF level: 55

Full MS
m/z range: 70-1000
Resolution: 70,000
AGC Target: 3e6
Maximum IT: 200ms
Polarity: positive and negative (polarity switching)

Data-dependent MS2:
Resolution: 17,500
AGC Target: 1e5
Maximum IT: 50ms
TopN: 10
Isolation Window: 0.4m/z
Normalized Collision energy: 35

Data Processing:
Non-targeted:
Software: Thermo Scientific Compound Discoverer 3.2
Databases: HMDB/KEGG/BioCyc, mzCloud
Ppm error window: 5ppm
Targeted: mevalonic acid (m/z 147.0663, negative mode)

Software: Thermo Scientific Xcalibur 4.5, Qual Browser
Ppm error window: 5ppm

Key resources

REAGENT or RESOURCE	SOURCE	IDENTIFIER	RRID
Antibodies			
Actin	Cell Signaling Technology	SC-4967S	AB_330288
DMT1/SLC11A2 (D3V8G)	Cell Signaling Technology	SC-15083	AB_2798699
GPX4	Cell Signaling Technology	SC-52455	AB_2924984
HMGCR	Abcam	ab242315	AB_2928124
xCT/SLC7A11 (D2M7A)	Cell Signaling Technology	SC-12691	AB_2687474
KEAP1 (D6B12)	Cell Signaling Technology	SC-8047	AB_10860776
MTTP	Abcam	AB75316	AB_1310473
Chemicals, peptides, and recombinant proteins			
Matrigel	Corning	354234	
BCA	Pierce Chemical Co. (Thermo Fisher Scientific)	23225	
ABC Reagent Kit	Vector Labs	PK-4000	
DAB chromogen	Vector LabsD	SK-4100	
PBS	Wisent	331-010-EL	
Antibiotic + antimycotic	Wisent	450-115-EL	
N2 Supplement	Gibco	17502-048	
Anti-rabbit HRP	Cell Signaling Technology	7074S	AB_2099233
Anti-mouse HRP	Cell Signaling Technology	7076S	AB_330924
DMSO	Sigma	276855	

Temozolomide	Sigma	T2577	
Lomitapide	Sigma	SML1385	
bFGF	Sigma	F0291	
EGF	Sigma	E9644	
DMEM	Wisent	319-005-CL	
DMEM F12	Wisent	319-075-CL	
B27	Sigma	17504044	
Penn strep	Wisent	450-201-EL	
L-Glut	Wisent	609-065-EL	
FBS	Wisent	920-040	
Trypsin/EDTA 0.05%	Wisent	325-542-EL	
Accutase solution	Sigma	A6964	
Puromycin	Sigma	P8833	
Neomycin	Sigma	N6386	
Complete mini EDTA-free protease inhibitor cocktail tablets	Roche	11836170001	
D-Luciferin	PerkinElmer	122799	
ECL kit	PerkinElmer	ORT2655	
Critical commercial assays			
CellTiter-Blue cell viability reagent	Promega	G8081	
	Abcam	AB113851	
Cellular ROS assay kit			
Cholesterol uptake assay kit	Abcam	AB236212	
Human Coenzyme Q_{10} (CoQ_{10})	Cusabio	CSB-E14081h	
ELISA	Abcam	AB102530	
Glutathione Peroxidase Assay Kit (Colorimetric)			
Experimental models: Cell lines			
U251 MG	Sigma	09063001	CVCL_0021
H818	Dr. Frederick Lang, M.D Anderson		

	(Houston, Texas)		
G811	Dr. Frederick Lang, M.D Anderson (Houston, Texas)		
GliNS1	Dr. Peter Dirks, Hospital for Sick Children (Toronto, Canada)		
Experimental models: Organisms/strains			
NOD *SCID* gamma mice	The Jackson Laboratory	005557	IMSR_JAX:005557
Software and algorithms			
PRISM	GraphPad		SCR_005375
ImageJ	NIH		SCR_003070
Halo	Indica Labs		SCR_018350
FlowJo	BD		SCR_008520
Affinity Designer	Serif		SCR_016952
