# Supplementary material for: Lomitapide enhances cytotoxic effects of temozolomide in chemotherapy-resistant glioblastoma
Source: JCI Insight. 2025 Jul 22;10(17):e186703. doi: 10.1172/jci.insight.186703 (PMC12487684; doi:10.1172/jci.insight.186703)

Full unedited gel for Figure 3A

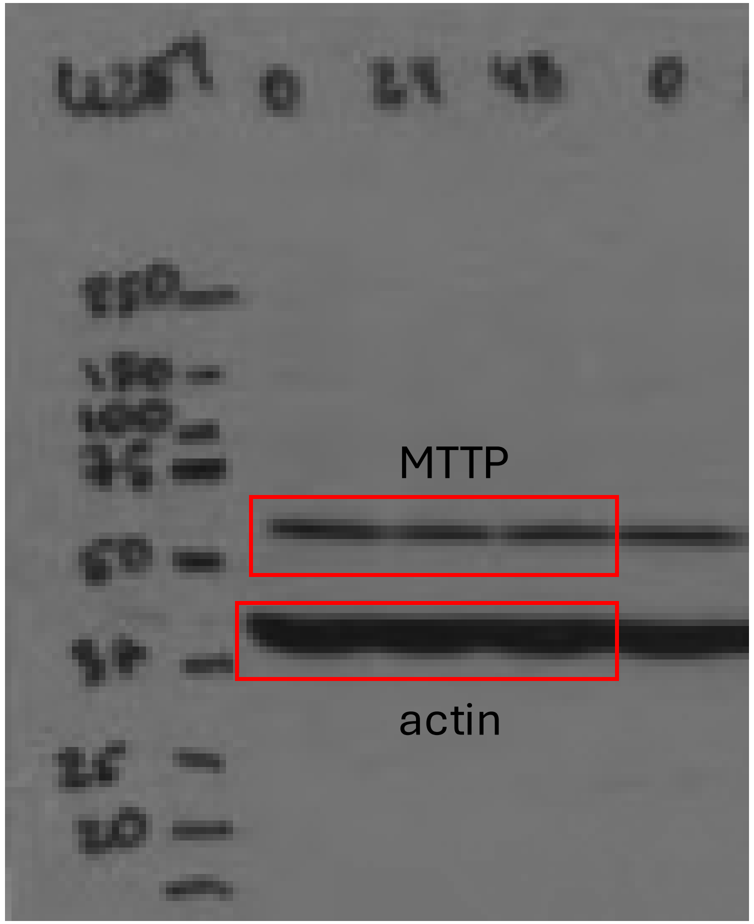

Full unedited gel for Figure 3C

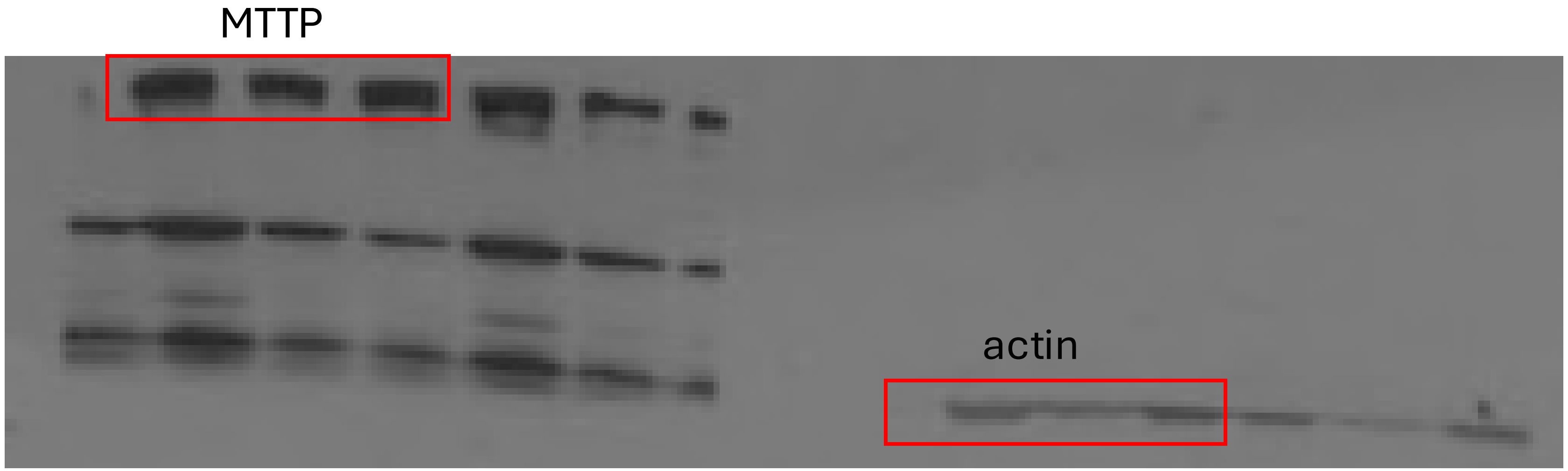

low exposure

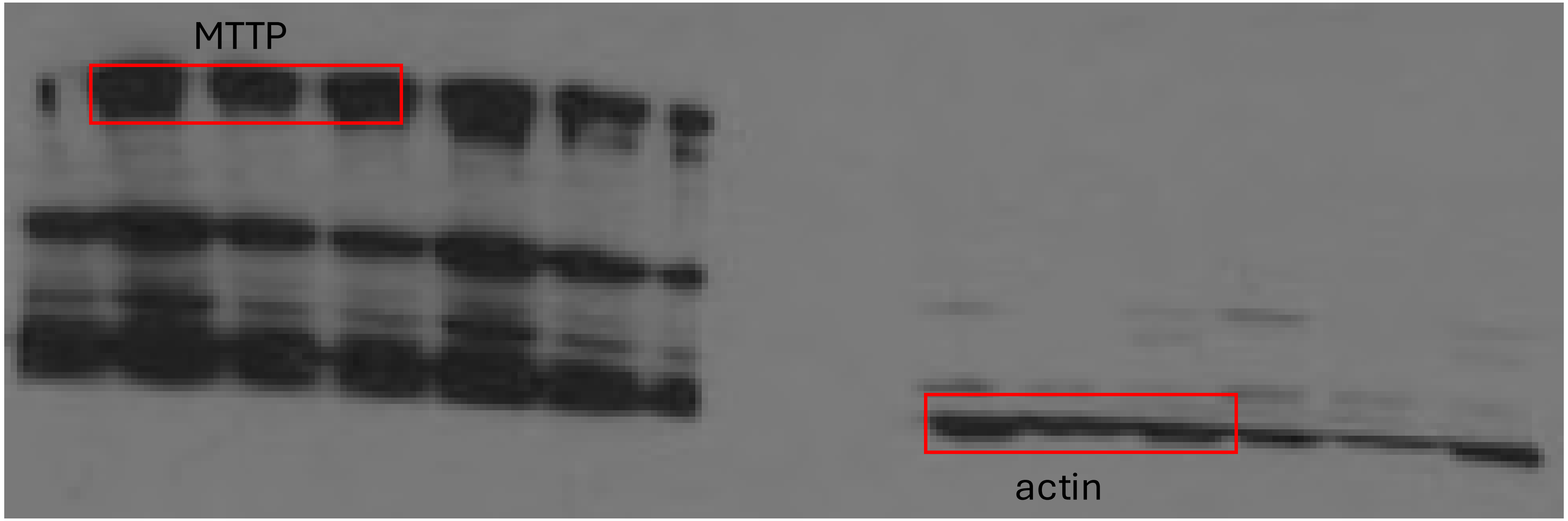

high exposure

Full unedited gel for Figure 3G

24 hr

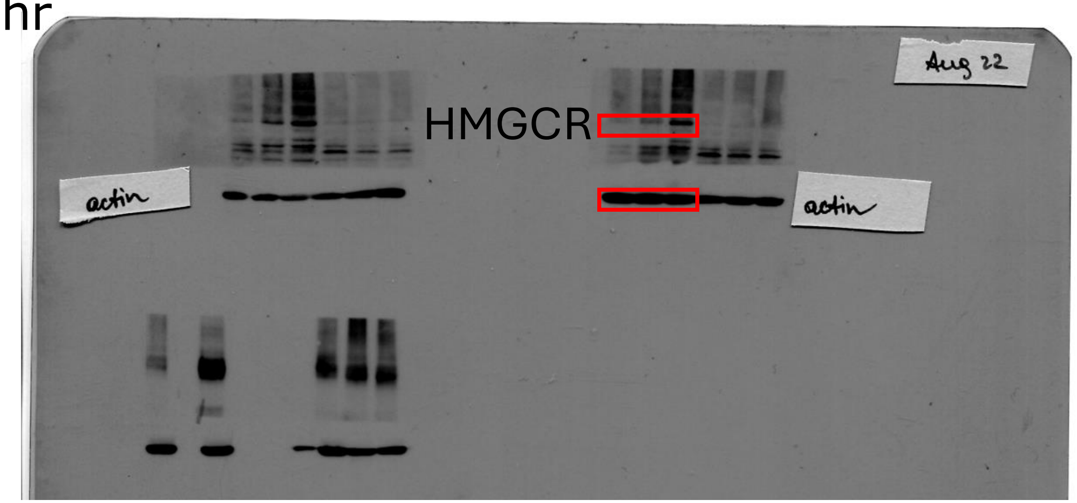

48 hr

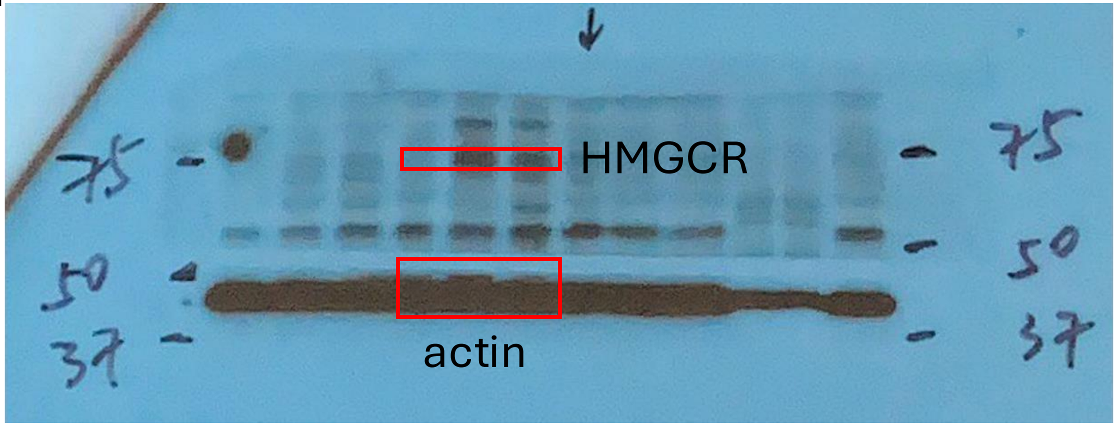

high exposure

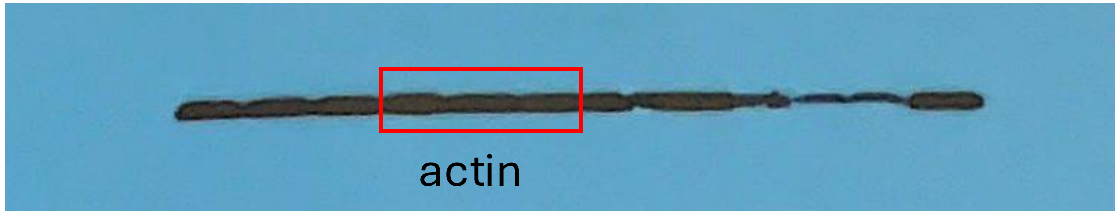

low exposure

Full unedited gel for Figure 3I

HMGCR

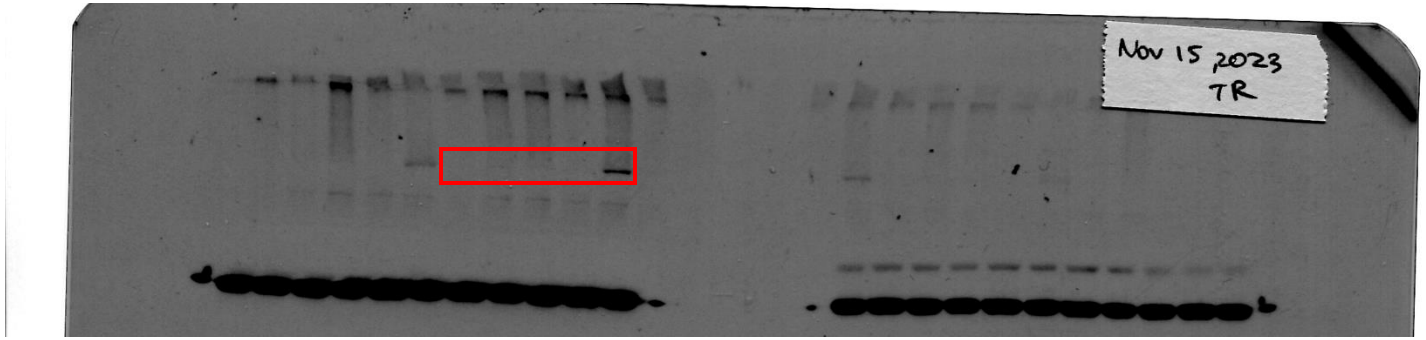

actin

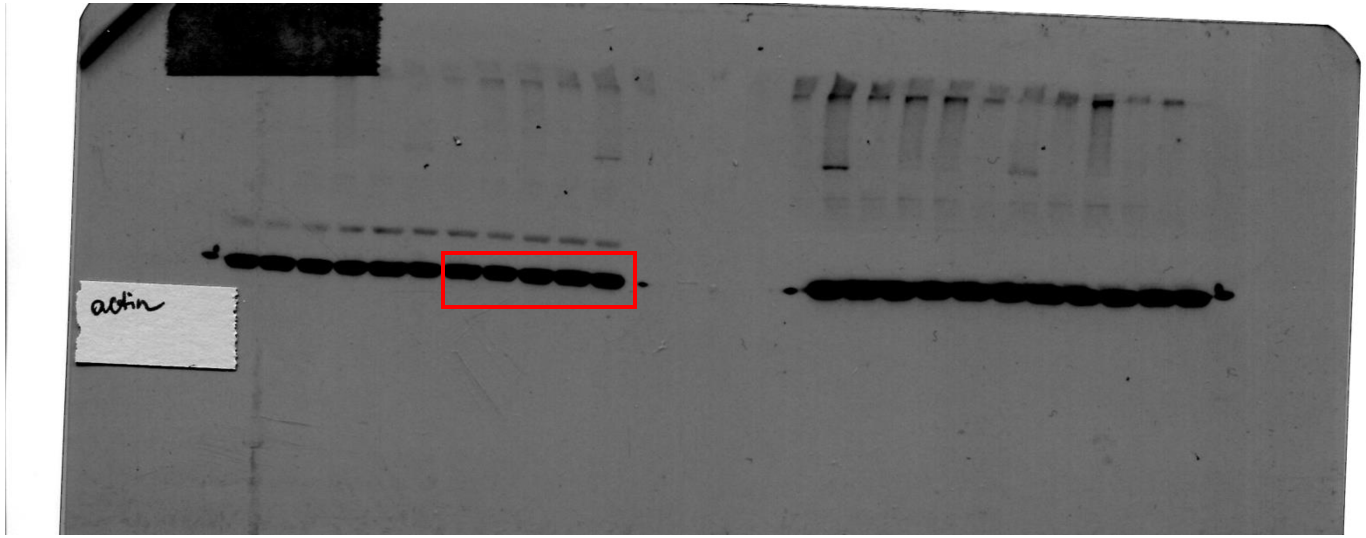

Full unedited gel for Figure 3K

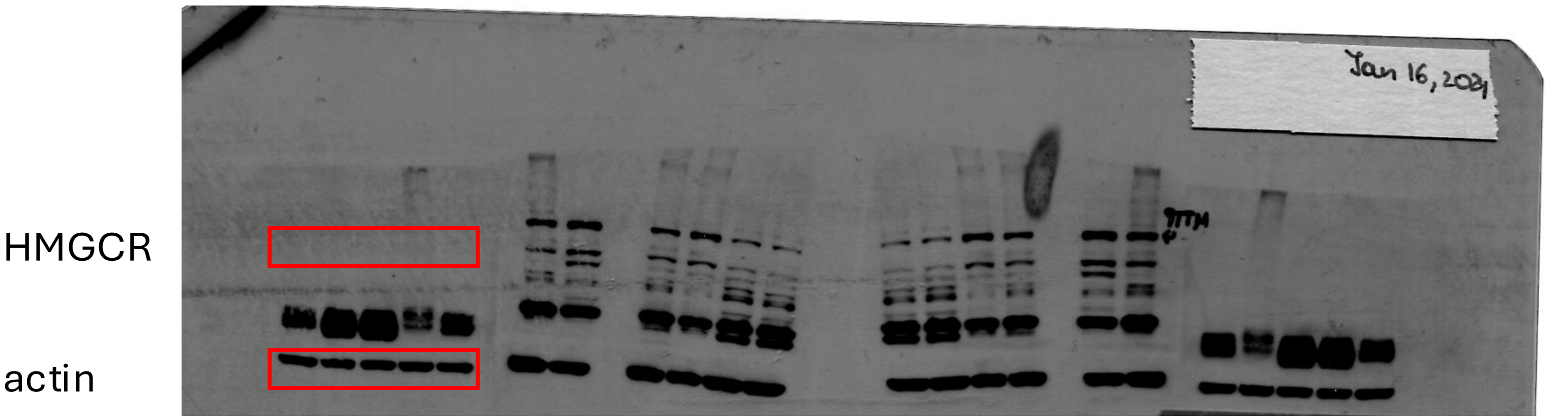

Full unedited gel for Figure 6

U251 cells

KEAP1

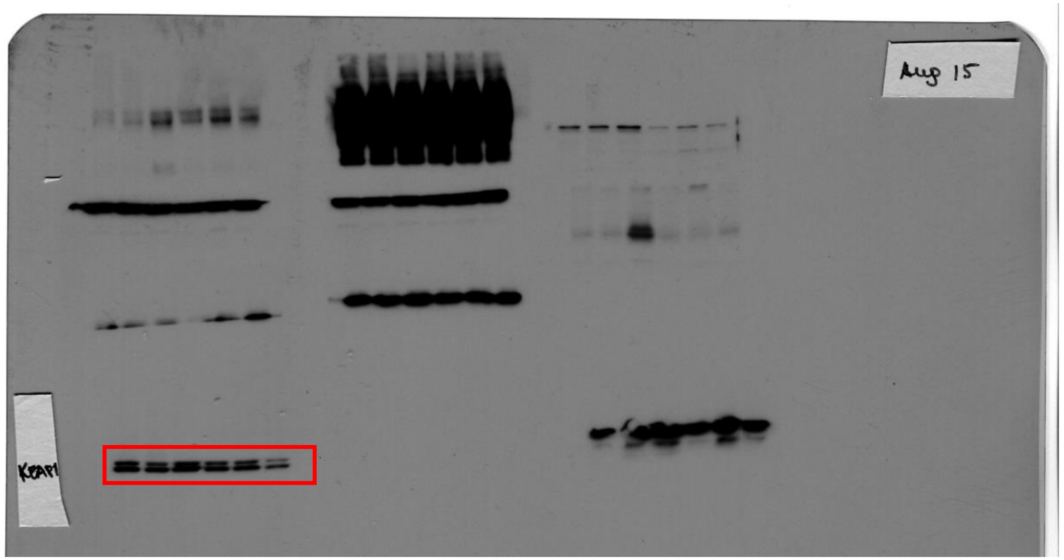

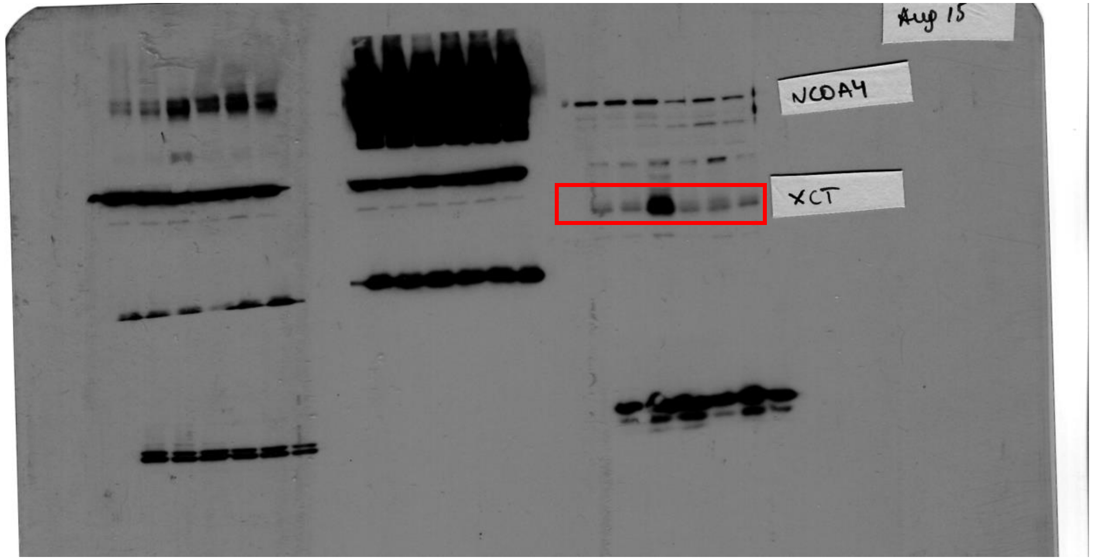

xCT

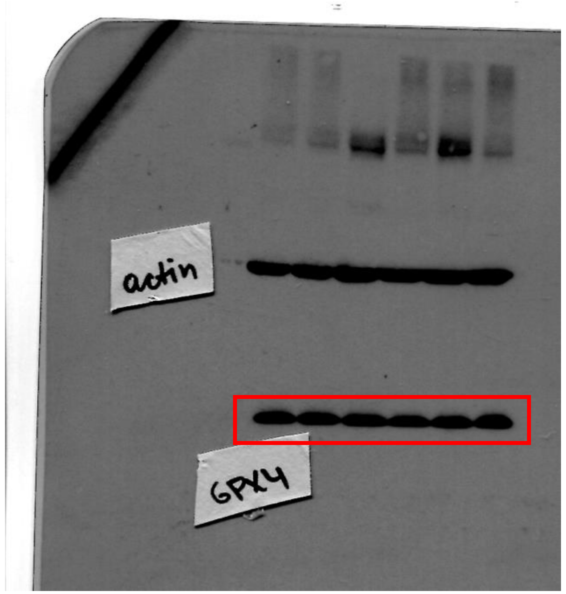

GPX4

DMT1

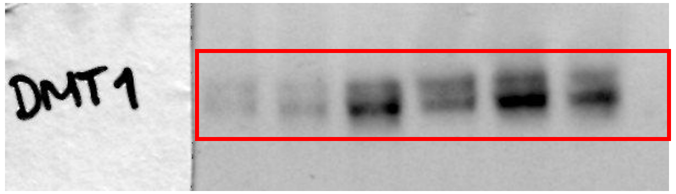

actin

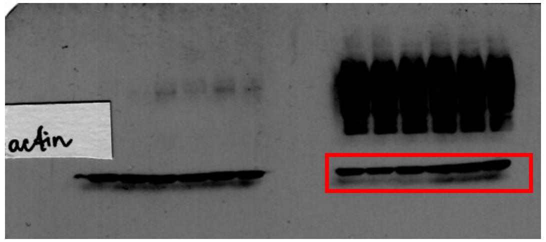

Full unedited gel for Figure 6K TR cells

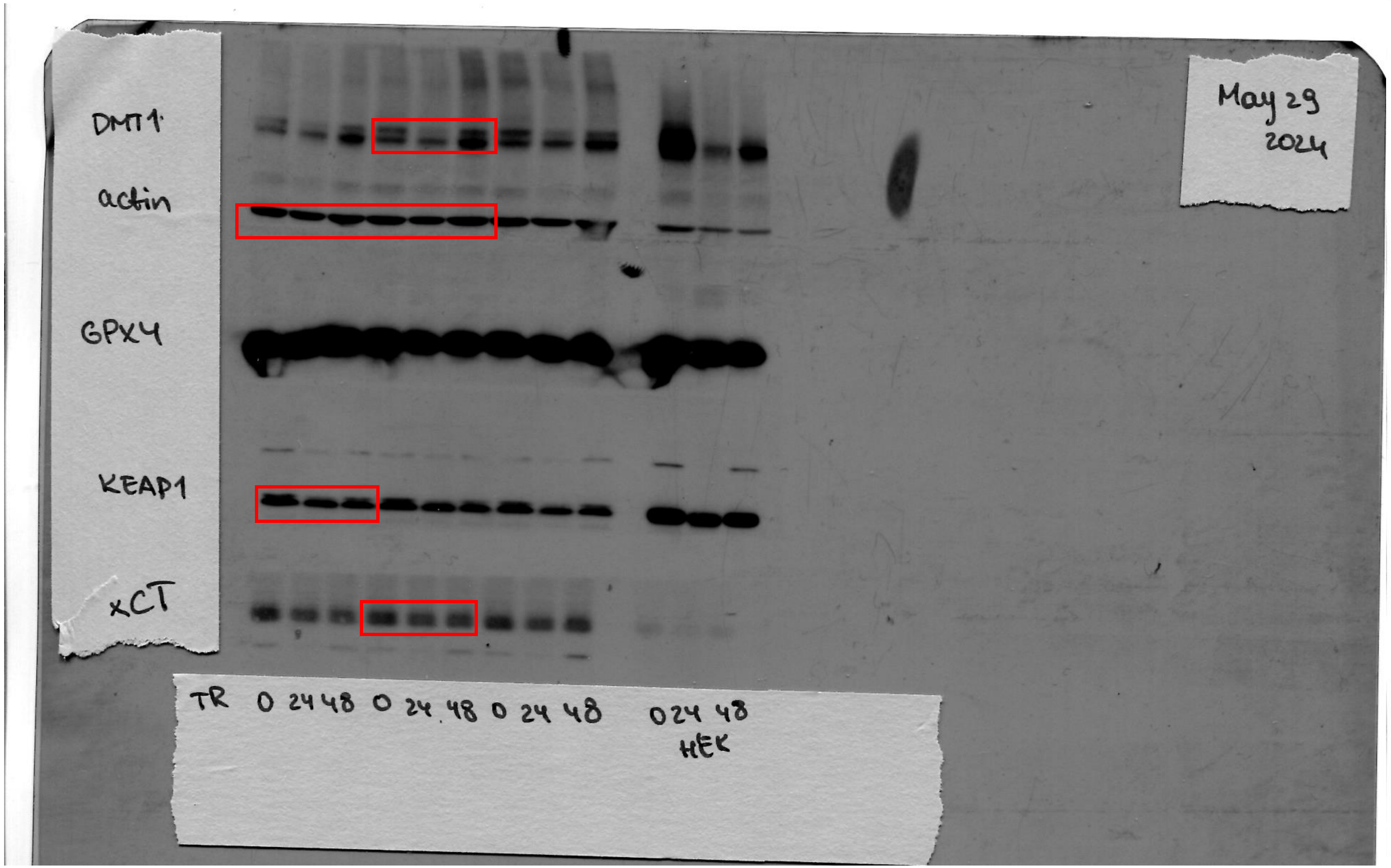

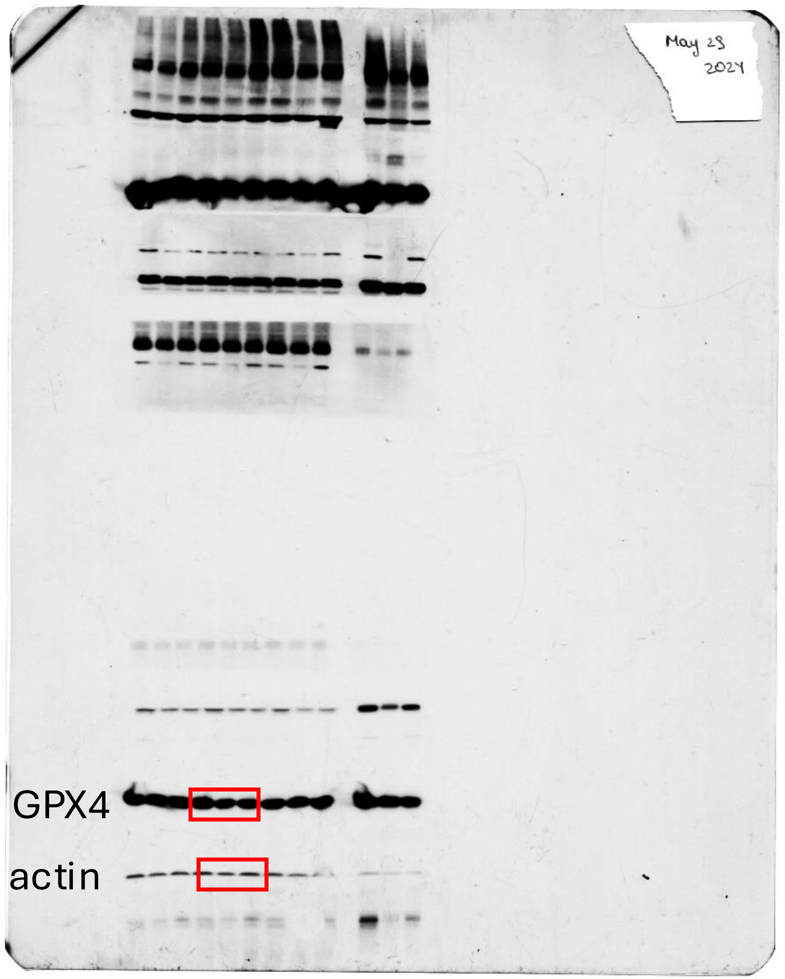

Full unedited gel for Figure 6L

HEK293 cells

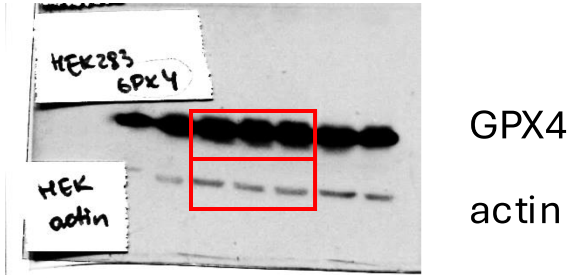

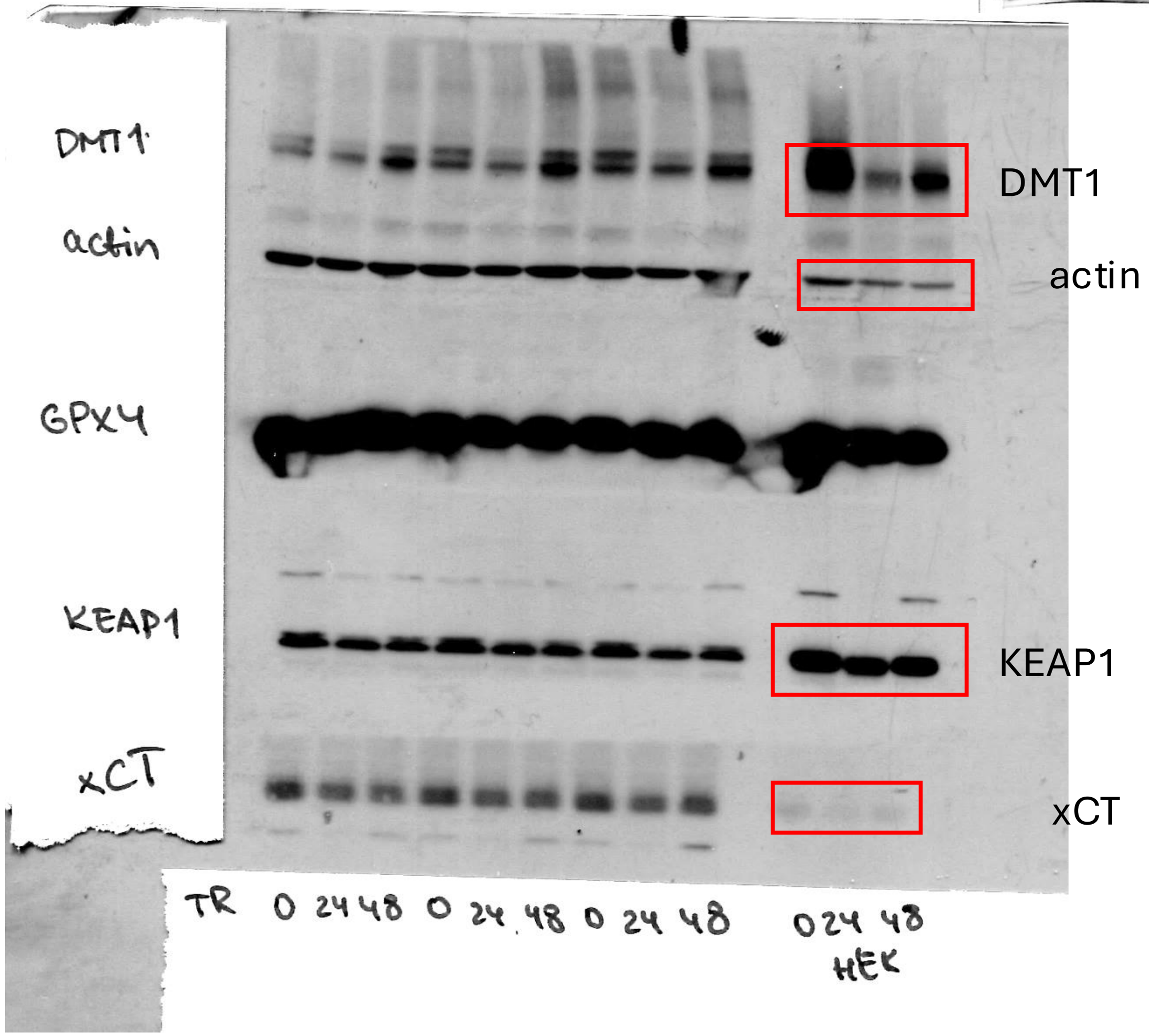

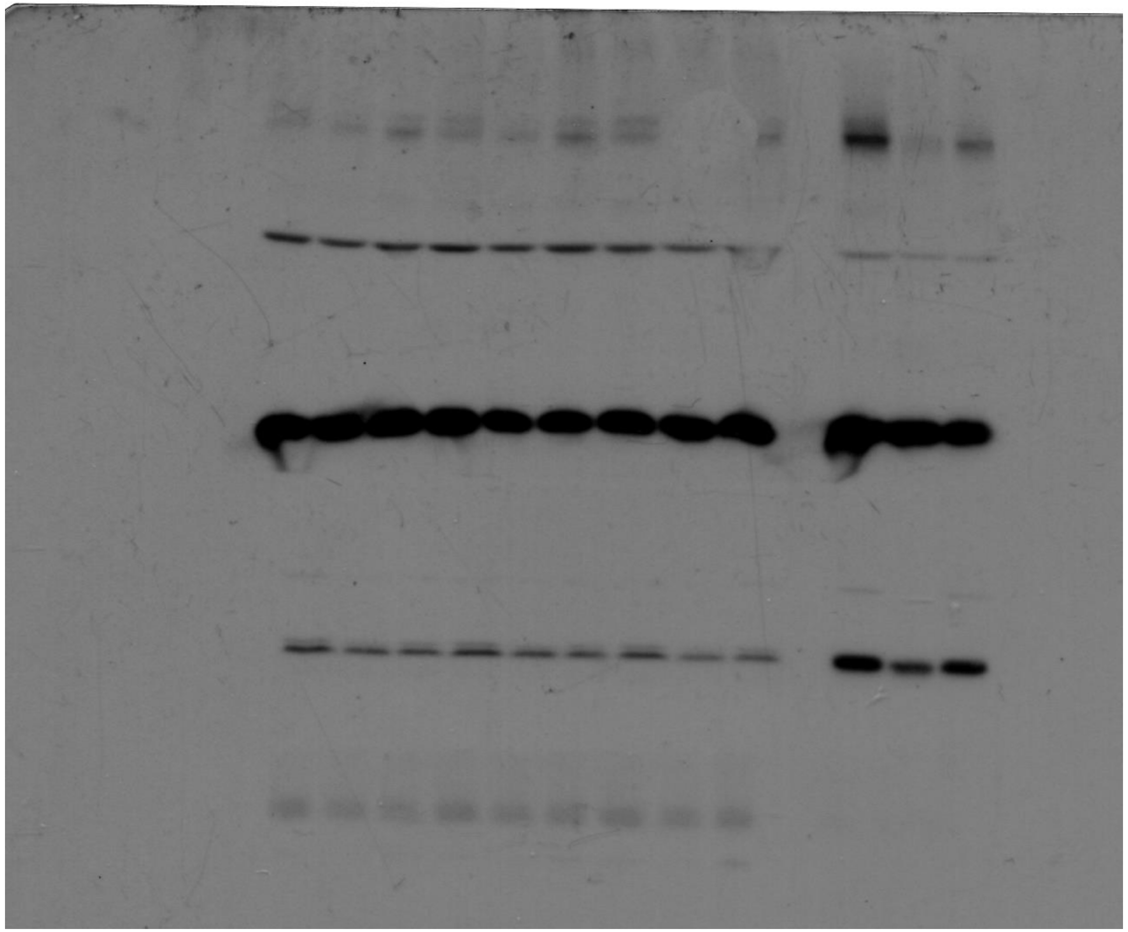

Supplement: Unedited blot and gel images [file jciinsight-10-186703-s180.pdf]
